# Indomethacin-incorporated microemulsion-laden contact lenses for improved ocular drug delivery and therapeutic efficacy†

**DOI:** 10.1039/d5ra01046b

**Published:** 2025-05-14

**Authors:** Kashvi Panchal, Yashkumar Patel, Harshilkumar Jani, Mittal Dalal, Vijay R. Chidrawar, Deepanjan Datta, Popat Mohite, Abhijeet Puri, Ketan Ranch, Sudarshan Singh

**Affiliations:** a Department of Pharmaceutics and Pharmaceutical Technology, L. M. College of Pharmacy Ahmedabad Gujarat 380009 India kashvi38@gmail.com ranchketan@gmail.com yash.patel@lmcp.ac.in harshil.jani@lmcp.ac.in; b Department of Pharmacology, L. M. College of Pharmacy Ahmedabad Gujarat 380009 India mittal.dalal@lmcp.ac.in; c School of Pharmacy and Technology Management, SVKM's Narsee Monjee Institute of Management Studies (NMIMS), Deemed-to-University Green Industrial Park, TSIIC, Jadcherla Hyderabad 509301 India vijay.chidrawar@gmail.com; d Department of Pharmaceutics, Manipal College of Pharmaceutical Sciences, Manipal Academy of Higher Education Manipal Karnataka 576104 India deepanjandtt@gmail.com; e AETs St. John Institute of Pharmacy and Research Palghar Maharashtra 401404 India mohitepb@gmail.com abhijeetp@sjipr.edu.in; f Office of Research Administration, Chiang Mai University Chiang Mai 50200 Thailand; g Faculty of Pharmacy, Chiang Mai University Chiang Mai 50200 Thailand sudarshan.s@cmu.ac.th

## Abstract

Conventional eye drops are associated with several limitations, including rapid drug clearance and low bioavailability, with only about 5% of the administered drug reaching the cornea to demonstrate therapeutic efficacy. Thus, to address these challenges, indomethacin (IND)-loaded microemulsion (Me)-embedded soft contact lenses (CLs) were developed to improve ocular drug delivery. The D-optimal mixture design was employed to optimize the composition of the Me formulation. Independent variables included Capmul MCM (oil phase), *S*_mix_ (Tween 80/isopropyl alcohol), and water, while dependent variables were the globule size, transmittance (%), and drug release profile. The optimized Me exhibited a globule diameter of 45.69 ± 1.85 nm and a transmittance of 99.4% ± 1.59%. The globule dispersion index (PDI) was 0.33 ± 0.06, and the zeta potential (ZP) was 0.657 ± 0.012 mV. Soft CLs were developed using free radical polymerization and fortified with the Me loaded with the drug through direct loading and soaking techniques. Direct loading achieved a swelling percentage of 96.37% ± 1.8% and a transmittance of 96.5% ± 0.3%, while soaking resulted in 97.57% ± 1.4% swelling and 97.3% ± 1.3% transmittance. A drug content of 19.76 ± 0.23 μg per lenses for direct loading and 29.8 ± 0.2 μg per lenses for soaking demonstrated that the efficacy of the soaking technique was higher than that of direct loading. Moreover, drug release studies showed that the Me-laden lenses prepared using the direct loading technique released 44.00% ± 0.53% to 53.00% ± 0.59% of the drug within the first 6 h, while the lenses prepared *via* soaking released 62.58% ± 1.56% to 97.64% ± 1.52% of the drug within the first 6 h, followed by regulated drug release for up to 28 h, maintaining clarity and drug loading. Ocular irritancy test results indicated negligible irritation, suggesting that the Me-laden lenses are a safe and effective platform to manage ocular inflammation with the controlled delivery of IND.

## Abstract 2

Conventional eye drops are associated with several limitations, including rapid drug clearance and low bioavailability, with only about 5% of the administered drug reaching the cornea to demonstrate therapeutic efficacy.

## Graphical abstract

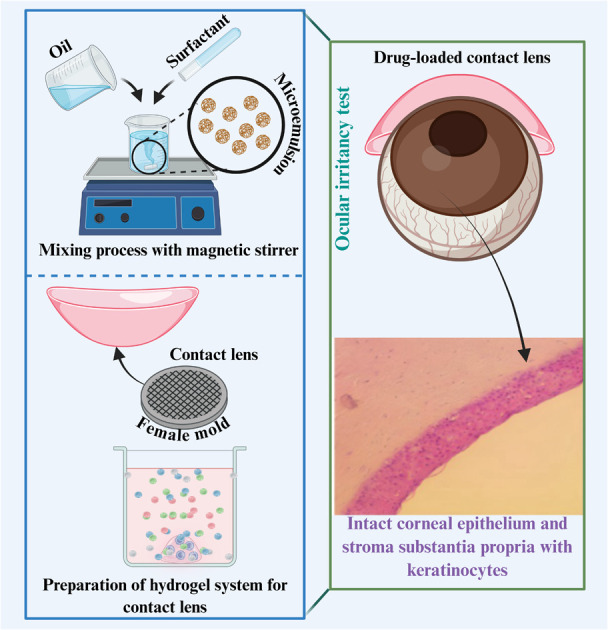

## 1 Introduction

Although conventional eye drops are a flexible form of ocular dosing, they frequently exhibit serious drawbacks, such as quick removal from the pre-corneal region as a result of lacrimation, tear dilution, and tear turnover.^1^ Additionally, the presence of tissue barriers, such as the conjunctiva, cornea, sclera, and lenses, further reduces the bioavailability of the drug in the cornea to about 5%, necessitating frequent administration to maintain therapeutic levels.^2^ These challenges underscore the need for alternative approaches to enhance the pre-corneal residence time and *trans*-corneal permeability for improving therapeutic outcomes.

The pre-corneal residence time and *trans*-corneal permeability are key parameters governing the effectiveness of the therapeutic regimen. Thus, several attempts have been made to address the challenges associated with conventional eye drops, including enhancing the pre-corneal residence time of drugs. These strategies involve the use of excipients that increase viscosity and/or mucoadhesion. From the perspective of drug delivery systems, semisolid formulations such as ointments (such as indomethacin eye ointment) and liquid formulations with higher viscosities have been fabricated. However, because their high viscosity causes blurred vision, they are mostly limited to night use.^3^ Alternatively, the use of nano-formulations, including polymeric nanoparticles,^4^ Me,^5^ liposomes,^6^ solid lipid nanoparticles (indomethacin),^7^ nanostructured lipid carrier indomethacin,^8^ and self-micro-emulsifying drug delivery systems^9^ incorporated in *in situ* gels can enhance the pre-corneal residence time and *trans*-corneal permeability. Solid formulations such as CLs can also accommodate nano-formulations to enhance the pre-corneal residence time.^10^

Microemulsions provide several benefits, such as improved biomaterial wettability, high drug-loading capacity, ease of production, and thermodynamic stability.^11^ For instance, researcher developed bimatoprost-loaded Me-laden CLs to treat glaucoma, demonstrating a two-fold improvement in drug uptake and sustained release profiles compared to conventional soaking techniques, without altering the critical lens properties.^11^ Similarly, Yanchu Li *et al.* studied the effect of Mes on ofloxacin uptake in CLs for treating conjunctivitis, showing the enhanced drug loading, release kinetics, and physical features of the lenses.^12^ In another study, Datta *et al.* and Ning Wei *et al.* developed cyclosporine-loaded microneedle CLs and timolol-loaded Me-laden silicone CLs, respectively, for glaucoma management, achieving enhanced drug loading and prolonged drug release without affecting the swelling and transmittance features of the lenses.^13,14^

To manage ocular inflammation conditions, corticosteroids (prednisolone acetate, dexamethasone, and triamcinolone acetonide) and non-steroidal anti-inflammatory drug (bromfenac, nepafenac, diclofenac, and indomethacin) are widely used.^15,16^ Indomethacin (IND) is a BCS class II drug with low aqueous solubility. As a topical non-steroidal anti-inflammatory drug, IND is commonly used to treat various ocular inflammatory conditions, including anterior segment inflammation,^7^ uveitis,^17^ conjunctivitis,^18^ macular edema,^19^ cystoid macular edema,^20^ and post-operative pain following cataract surgery.^21^ This drug works by inhibiting cyclooxygenase enzymes and blocking prostaglandin synthesis, thereby reducing inflammation.^22^ Topical administration, especially *via* eye drops, is the favored technique for delivering IND to the eye due to its simplicity, minimal systemic side effects, and high patient compliance.^23,24^ Eye drops are the major delivery route for treating anterior segment diseases such as inflammation due to their ability to target the site directly.^7^ However, drawbacks including frequent instillation, rapid clearance and loss due to nasolacrimal drainage lead to low ocular bioavailability and patient non-compliance when IND is delivered *via* the ophthalmic route. Alternatively, IND-Me addresses the low aqueous solubility issue and enhances the solubility of IND, which can accommodate a greater drug content in Me compared to eye drops. However, the short residence time of Me is still an issue; hence, in this study, we incorporated Me into CLs without compromising the critical quality attributes of the lenses, which can affect patient compliance. The Me-loaded CLs can accommodate IND above the daily therapeutic dose, which can solve the frequent instillation, and thus increase patient compliance. This strategy can be beneficial for delivering the BCS class II and class IV drugs to the eye in a sustained manner.

These studies emphasize the potential of Me-loaded CLs as a superior drug delivery system, offering enhanced drug uptake, sustained release, and improved retention compared to conventional techniques. Consequently, the research objective was to develop and optimize IND-Me to enhance the drug solubility and loading. The optimized IN-Me, integrated into soft CLs for ocular drug delivery, aimed to enhance the bioavailability, prolong the drug release, and preserve the fundamental physical features of the CLs. The success of this approach can provide a valuable solution for improving the treatment of ocular inflammatory disorders, particularly where conventional eye drop formulations fall short.

## 2 Materials and methods

Indomethacin (*M*_w_: 357.787 g mol^−1^; > 98%) was provided as a gift sample from Cadila Healthcare Ltd, Gujarat, India. Hydroxyethyl methacrylate (HEMA; 130.14 g mol^−1^), isopropyl myristate (0.85 g cm^−3^), polyethylene glycol 400 (1.13 g cm^−3^), propylene glycol (1.04 g cm^−3^), isopropyl alcohol (786 kg m^−3^), triethanolamine oleate (1.0 g cm^−3^), polysorbate 20 (1.1 g cm^−3^), polysorbate 60 (1.08 g cm^−3^), polysorbate 80 (1.06 g cm^−3^), sorbitan mono-oleate (0.986 g cm^−3^), sorbitan trioleate (0.956 g cm^−3^), sorbitan monolaurate (1.032 g cm^−3^) and methacrylic acid (MAA; 1.02 g cm^−3^) were procured from Sigma-Aldrich, Massachusetts, United States. Ethylene glycol di-methacrylate (EGDMA; 198.22 g mol^−1^) and diphenyl(2,4,6-trimethylbenzoyl)phosphine oxide (Duracur®) were obtained from Tokyo Chemicals (India) Pvt. Ltd, Hyderabad, India. Octadec-9-enoic acid (0.88 g cm^−3^) was acquired from S.D. Fine Chemicals, Gujarat, India. Propylene glycol dicaprylate (Captex 200) (0.929 g cm^−3^), triglycerides of caprylic/capric acid (Captex 355) (0.960 g cm^−3^), and glyceryl monocaprylate (Capmul MCM) (0.950 g cm^−3^) were purchased from Abitec Corporation. Octanoic acid (Caprylic oil) (0.91 g cm^−3^) and PEG-8 caprylic/capric glycerides (Mayasol) were obtained from Subhash Chemical Industries, Maharashtra, India. Polyoxyethylene glycol (Lutrol F68) (1.14 g cm^−3^) was sourced from Signet Chemical Corporation Pvt. Ltd, Maharashtra, India. Caprylocaproyl polyoxyl-8 glycerides (Labrasol ALF), polyglycol mono- and di-esters of 12-hydroxystearic acid 30% PEG (Solutol HS 515), and oleoyl polyoxyl-6-glycerides (Labrafil M 1944 CS) were procured from Gattefossé SAS. All other reagents were of analytical grade.

### 2.1. Screening of oil, surfactant, and cosurfactant

The solubility of indole-3-carbinol (IND) in a wide range of oils was evaluated. These oils included isopropyl myristate, oleic acid, Captex 200, Captex 355, Capmul MCM, caprylic oil, Labrafac PG, and Labrafil M 1944 CS, and surfactants such as Span 20, Tween 20, Span 80, Span 85, Tween 80, Tween 60, Lutrol F68, Labrasol ALF, Solutol HS 515, and Mayasol. Furthermore, cosurfactants such as PEG 400, propylene glycol, isopropyl alcohol, triethanolamine oleate, and PEG-7 glyceryl cocoate were also considered in the assessment.^25^ Excess IND was added to each type of oil, surfactant, and cosurfactant in sealed glass vials. Subsequently, the saturated solutions were agitated in an orbital shaker (Remi CIS-18 Plus, Germany) at 100 rpm and 37 °C ± 1 °C for day. Upon reaching equilibrium, the compositions were centrifuged at 8000 rpm for 20 min (cooling microcentrifuge; Sorvall Legend X1; Thermo Scientific; USA). Then, the supernatant was then, and its IND concentration was determined using UV-visible spectroscopy (Shimadzu UV-1900, Japan). The density of the oil was considered, and a linear equation (*y* = 0.0279*x* + 0.0144; *r*^2^ = 0.997) was obtained from the calibration curve (detailed data are presented in ESI File; Tables S1, S2, Fig. S1 and S2†). The components exhibiting high solubility for IND were selected to develop the pseudo-ternary phase diagram.

### 2.2. Construction of pseudo-ternary phase diagrams

The water titration process was employed to construct pseudo-ternary phase diagrams that delineate the concentration range of components within the Me region.^25,26^ Four phase diagrams were created by altering the surfactant (Tween 80) to cosurfactant (isopropyl alcohol) ratios as 1 : 1, 2 : 1, 1 : 2, and 4 : 1. Subsequently, the oil phase (Capmul MCM) was combined with the surfactant mixture in weight ratios ranging from 10 : 0 to 0 : 10. These mixtures were gradually diluted with deionized water while being stirred moderately on a magnetic stirrer at 50 rpm and ambient temperature. Subsequently, water was added dropwise until turbidity or phase separation occurred. The point at which the mixture turned turbid and biphasic indicated the termination of water addition. Transparent, single-phase systems were classified as Mes, which are characterized by low viscosity and a clear appearance. The critical points between the Me region and other phase regions were quantified by the transition from a clear state to a turbid one and *vice versa*. The quantity of water used was recorded to calculate the final percentages of water, oil, and surfactant/cosurfactant. Subsequently, these percentages were used to construct the pseudo-ternary phase diagrams. The results obtained from the water titration technique were exported to ProSim version 1.9, a program specifically designed for constructing pseudo-ternary phase diagrams.

### 2.3. Preparation of Me

Indomethacin-loaded Me formulations were developed using a spontaneous emulsification technique, as previously reported.^27^ Initially, Capmul MCM (oil), Tween 80 (surfactant), and isopropyl alcohol (co-surfactant) were thoroughly blended with magnetic stirring at 37 °C ± 2 °C. Subsequently, IND was admixed in this mixture, and deionized water was slowly added dropwise with vigorous vortexing for 1 to 2 min to achieve a uniform blend. Notably, no precipitates were evident in the final drug-loaded formulations. Post-preparation of microemulsion purification was done by means of filtration, as reported previously in the literature.^28^ The microemulsion was passed through a disposable syringe filter (0.22 μm Millipore filter) into 10 mL glass crew capped vials. Not more than 10 mL of microemulsion was filtered through each disposable syringe filter.

### 2.4. Optimization of indomethacin Me using D-optimal mixture design

The D-optimal mixture design was utilized to optimize the compositions of the Me formulations. After identifying the Me region from the pseudo-ternary phase diagram, batches were prepared using the D-optimal design approach. Specifically, variations in the levels of Capmul MCM (oil phase), the ratio of Tween 80 to isopropyl alcohol (surfactant–cosurfactant combination, 2 : 1), and deionized water were selected within the Me region. The response varying for these batches included globule size (Y1 in nm), percent transmittance of diluted Me (Y2), and percent drug release after 6 h from Me-laden CLs prepared *via* direct loading. The experimental design, together with the predefined levels, was implemented using the Design-Expert software version 12 (State-Ease Inc., Minneapolis, Minnesota, USA) to create the design space. The independent and dependent variables used in the D-optimal mixture design are listed in Table 1. The composition of oil, *S*_mix_, and water is presented in Table S3.†

D-optimal mixture design

**Table:** 

Independent variables					
X1		X2		X3	
Oil phase (Capmul MCM) (mL)		*S*_mix_ (Tween 80/Iso propyl alcohol) (2 : 1) (mL)		Water (mL)	
Low	High	Low	High	Low	High
0.100	0.550	0.350	0.800	0.100	0.550

**Table:** 

Dependent variables		
Y1	Y2	Y3
Globule size (nm)	Transmittance (%) of Me diluted with water	Percentage drug release after 6 h from Me-laden CLs prepared using the direct loading technique

### 2.5. Characterization of Mes

#### 2.5.1. Dilution and transmittance

A dilution test was performed to assess the miscibility and stability of the IND-loaded Me with its external phase. To determine the Me type, the IND-loaded formulation was subjected to dilution and observed for any physical changes. The percentage transmittance of Me was further assessed using UV spectroscopy (Shimadzu UV-1900 Spectrophotometer, Japan) at 630 nm against deionized water as the reference, as previously reported.^29^ The Me batches were diluted up to 10-fold with deionized water, and the transmittance (%) at 630 nm was recorded. The ability of Me to maintain transparency upon dilution reflected its stability under varying conditions, confirming the oil-in-water (o/w) nature of the formulation.

#### 2.5.2. Globule size, polydispersity index, ZP, and morphology

The globule size, polydispersity index (PDI), and ZP of the Me batches were measured using a Malvern Zetasizer NanoZS-90 (Malvern Instruments, USA), employing dynamic light scattering (DLS), as described in the literature.^30^ Each Me sample was diluted ten-fold with deionized water and sonicated using an ultrasonic cleaner (LMU6, LABMAN, Mumbai, India) to ensure homogeneity. To determine globule size and PDI, the sample was placed in a disposable cuvette (Malvern Pan-Analytical), and measurements were recorded at a 90° scattering angle and 25 °C. PDI was used to evaluate the size distribution within the Me, while globule size and PDI measurements were performed to identify any aggregates that might impact the optical clarity of the lenses. Furthermore, the ZP was measured for 1 mL of diluted Me using a Malvern Zetasizer, followed by surface charge quantification under the same temperature conditions. All analyses were performed in triplicate (*n* = 3, mean ± SD). Furthermore, based on the size results, formulation runs 6 and 12 were subjected to morphology analysis using transmission electron microscopy, as reported. In brief, a drop of Me was deposited on a cooper grid and allowed to rest. Later, the excess fluid was gently drained from the grid. Subsequently, a negative staining solution (phosphotungstic acid 1%) was applied dropwise. Finally, the grid was air dried and examined for morphology under a transmission electron microscope at an operative voltage of 80 kV and magnification of 350×.

#### 2.5.3. Thermodynamic stability

The thermodynamic stability of Me was evaluated to assess its long-term physical stability under the reported storage conditions.^31^ Mes was centrifuged at 10 000 rpm for 30 min to check for any phase inversion/separation or cracking. The samples that showed signs of instability were excluded from further analysis. The stable Mes were subjected to a freeze–thaw cycle for 48 h, involving both heating and cooling phases. In the heating phase, the Mes were heated at 45 °C for 48 h, followed by cooling at 4 °C. Throughout these cycles, any phase separation, cracking, or creaming was carefully observed. The Mes that remained stable throughout all tests were deemed thermodynamically stable and suitable for further use.

### 2.6. Fabrication of hydrogel-based CLs

Hydrogel CLs were developed using the free radical polymerization technique, as reported in the literature.^32^ The previously reported pre-monomer mixture composed of 46.7% (w/w) of HEMA, 0.8% (w/w) of MAA, 1% (w/w) of EGDMA, and 52% (w/w) of water was utilized for this study.^32^ This mixture yielded hydrogel-based CLs of desirable and adequate quality considering swelling, transparency and strength. To ensure thorough mixing and removal of air bubbles, the monomer mixture was sonicated for 10 min in a bath sonicator (LMU6, LAB MAN, Mumbai, India). Following sonication, 50 μL of the pre-monomer mixture was dispensed onto a female mould using a micropipette. Subsequently, a male mould was positioned over the female mould to establish the lens shape. The assembled moulds were exposed to 366 nm ultraviolet (UV) radiation from a UV cabinet (CLE-UV by Coral Labtech Enterprises, Vapi, India) for a duration of 20 min to initiate and complete the polymerization process of the hydrogel lenses. After curing, the lenses were carefully removed from the moulds and placed in vials containing 2 mL of simulated tear fluid (STF; pH 7.4) as the storage solution.

### 2.7. Loading indomethacin into CLs

The pure IND and IND-loaded Mes were incorporated into CLs using two techniques, *i.e.*, the conventional soaking technique and the conventional direct loading technique. In the soaking technique, blank CLs were immersed in 10 mL of STF containing 10 mg of IND, or in a mixture of 2 mL IND-loaded Me and 8 mL STF for 24 h. In the direct loading technique, a drug suspension with 1 mg per mL IND or 1 mL of IND-loaded Me (1 mg mL^−1^) was added directly to the pre-monomer mixture during the fabrication of the lenses. Then the lenses were cured using UV light at 366 nm for 20 min to complete polymerization, in which the monomers were converted to polymers. These approaches enable efficient drug loading into CLs for potential ocular applications.^11,12^ The composition of Me in each CL loaded using the soaking technique (SM-ME-CL) and direct loading technique (DL-ME-CL) is shown in Table 2.

**Table 2:** Me-laden CLs prepared using the soaking and direct loading techniques

Microemulsion-laden CLs prepared by soaking technique	Composition of microemulsion in each Me-laden CLs		
	Oil (mL)	*S*_mix_ (2 : 1) (Tween 80/Isopropyl alcohol) (mL)	Water (mL)
ME-CL-1	0.229	0.535	0.235
ME-CL-2	0.322	0.572	0.104
ME-CL-3	0.323	0.350	0.326
ME-CL-4	0.437	0.350	0.212
ME-CL-5	0.550	0.350	0.100
ME-CL-6	0.100	0.573	0.326
ME-CL-7	0.322	0.572	0.104
ME-CL-8	0.436	0.463	0.100
ME-CL-9	0.173	0.426	0.400
ME-CL-10	0.100	0.350	0.550
ME-CL-11	0.229	0.535	0.235
ME-CL-12	0.100	0.800	0.100
ME-CL-13	0.100	0.350	0.550
ME-CL-14	0.100	0.573	0.326
ME-CL-15	0.323	0.350	0.326
ME-CL-16	0.125	0.676	0.197

### 2.8. Characterization of Me-laden contact lens

#### 2.8.1. Swelling and transmittance

Swelling studies were performed to assess the hydration level of the CLs using the previously reported technique.^33^ After fabricating the CLs, the dry lenses were carefully removed from the mould, and their dry weight (Wd) was recorded. Then, the lenses were immersed in 2 mL of packaging solution (STF, pH 7.4) and left to hydrate for 24 h at room temperature. Following hydration, the lenses were gently blotted with tissue paper to remove any excess moisture, and their swollen weight (*W*_s_) was measured.1
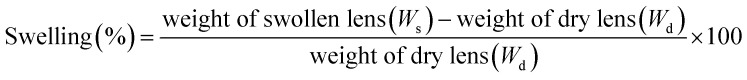


The optical transmittance of the CLs should ideally remain unaffected after the incorporation of Me. As reported, the transmittance of the Me-loaded CLs was evaluated using a UV spectrophotometer.^34^ After soaking the hydrated CLs in STF at pH 7.4 for 24 h, the lenses were placed in a quartz cuvette. Then, the cuvette was inserted into a UV-vis spectrophotometer, where the transmittance was recorded in the wavelength range of 200–800 nm.

#### 2.8.2. Determination of indomethacin content in CLs

The drug content (IND) in the Me-loaded CLs prepared using both the direct loading and soaking techniques was measured using ultraviolet spectroscopy, as described previously.^35^ To determine the drug concentration in the CLs, the lenses were cut into small pieces and soaked in 5 mL of methanol. Subsequently, the samples were agitated on a magnetic stirrer (specify the model, manufacturer, origin, and country) at 150 rpm for 24 h at room temperature to ensure complete extraction of the drug from the lenses. Finally, the methanol solution was analysed for drug content using a UV-vis spectrophotometer.

#### 2.8.3. Indomethacin leaching during sterilization study

The CLs were sterilized to avoid ocular irritation and inflammation, which can result in patient non-compliance.^36^ This test was aimed to quantify the quantity of IND leached from the Me-loaded CLs into the packaging solution during sterilization. The leaching of IND during sterilization was tested as reported.^37^ In brief, the hydrogel CLs were sterilized by autoclaving (Equitron SLE series autoclave by Medica Instrument Mfg. Co. Mumbai, Maharashtra, India) them in 2 mL of STF at 121 °C and 15 psi for 30 min. After sterilization, the packaging solution was analysed to measure the quantity of leached IND. The indomethacin levels in the packaging solution (STF) were quantified using UV spectroscopy.

#### 2.8.4. *In vitro* drug release of indomethacin Me-loaded CLs

*In vitro* drug release studies were performed on sterile CLs.^36^ Briefly, the CLs were placed in glass vials containing 2 mL of STF and maintained at 32 °C ± 1 °C on an orbital shaker incubator (CIS-18 Plus, REMI Electronics, Germany) set to shake at 100 rpm. This setup simulated *in vivo* tear turnover by keeping the dissolution medium volume constant at 2 mL. To assess drug release from the lenses, 2 mL of STF was withdrawn at predetermined intervals. To maintain sink conditions, the withdrawn STF was immediately restored with an equal quantity of fresh STF. The concentration of IND was measured using ultraviolet spectroscopy, and the drug release profile was assessed by plotting the cumulative drug release (%) over time.

#### 2.8.5. Ocular irritancy test

The *in vitro* ocular irritancy test was conducted using excised goat corneas, following an adapted procedure from OECD TG 437.^38^ The isolated goat cornea model is widely used for ocular irritancy testing due to its structural and biochemical similarities to human corneas.^39^ Goat corneas share comparable anatomy and physiology with human corneas, making them suitable for the initial assessment of ocular irritation.^39^ Furthermore, the use of isolated animal corneas reduces the ethical issues associated with live animal testing. Goat corneas were obtained from a local slaughterhouse (Al-Waris Chicken Center, Shop No. 6, Zetun Residency, TP 85 Rd, opp. Emaad Flat, opp. Amber tower, Fatehwadi, Arshad Park, Sarkhej, Ahmedabad, Gujarat, India) within one hour of the sacrificing the animals and stored in Hank's balanced salt solution at 2–8 °C until use. Each cornea was removed and exposed to the negative control (0.9% w/v NaCl saline solution), the test formulation (CLs), and the positive control (100% ethanol) for 10 ± 1 min. During exposure, the epithelial side of the cornea was treated with the respective solutions, while the opposite side was kept in contact with minimum essential medium to maintain the integrity of the cornea. After exposure, the corneas were rinsed with Hank's balanced salt solution, and corneal opacity was assessed using the Draize scoring technique. The presence of opacity was used as an indicator of irritancy or toxicity, with scores ranging from 0 (no opacity) to 4 (opaque), according to Blazka and Hayes' technique. After the opacity assessment, the corneas were preserved in 10% neutral formalin for 24 h, embedded in paraffin blocks, and sectioned for histopathological examination. The slides were stained with Hematoxylin and Eosin and analysed under a microscope to identify any morphological or histological alterations in the corneal tissue layers, offering insights into potential irritation or damage caused by the formulations, as documented.^40^

## 3 Results and discussion

The present work aimed to enhance the ocular drug delivery of IND using Me-laded CLs. Me can accommodate a higher amount of IND compared to conventional eye drops. We developed and optimized IND-Me using the D-optimal design. This Me was loaded in CLs using two different loading techniques. Subsequently, Me-laden CLs were further evaluated for their critical quality attributes.

### 3.1. Solubility study of indomethacin

**Fig. 1:**
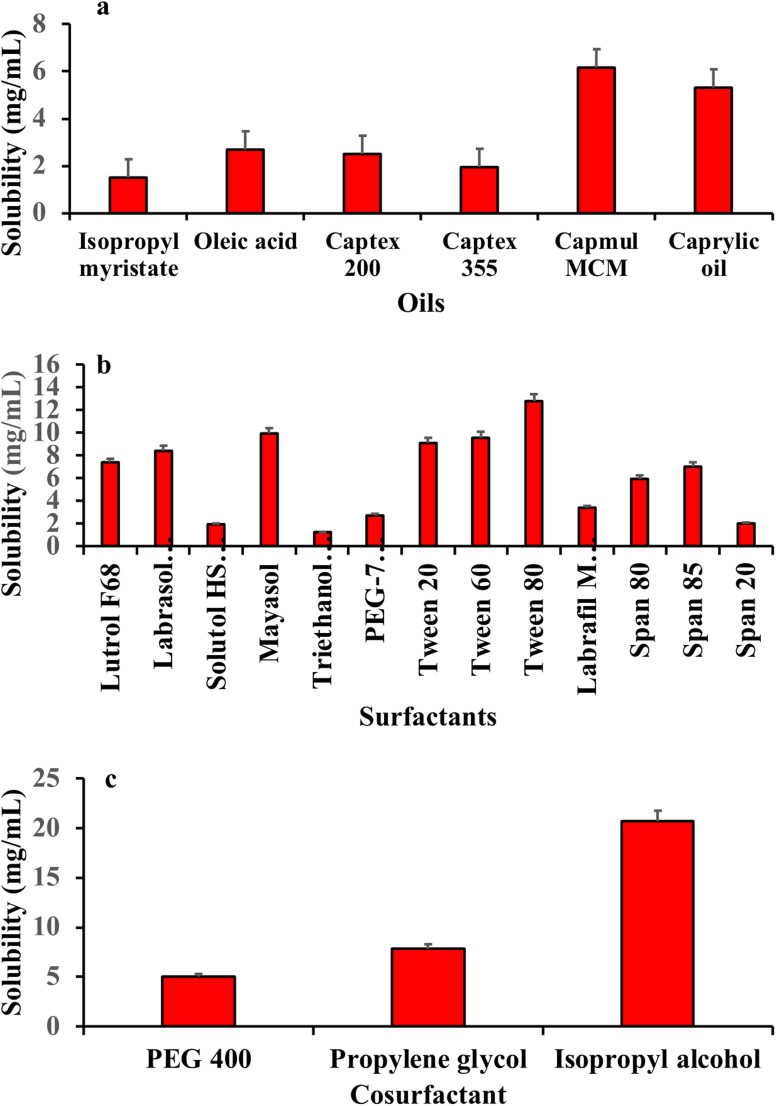
Solubility study of indomethacin in oil (a), surfactants (b) and co-surfactants (c).

The screening of the oil phase is essential in Me formulations to ensure the maximum drug loading, prevent drug precipitation, and improve the stability of the Me effects on droplet size and system stability. The solubility study was conducted employing the established methodology to identify suitable oils, surfactants, and co-surfactants. The findings revealed that among the tested excipients, Capmul MCM exhibited the highest solubility for IND in the oil phase. Tween 80 was selected as the surfactant, and isopropyl alcohol was chosen as the co-surfactant, as illustrated in Fig. 1a–c. Capmul MCM functions as both a solubilizer and an emulsifier. The ability of Capmul MCM to stabilize the emulsion enhanced the dispersion of IND in the oil phase, thereby increasing its solubility. The reduction in surface tension using Tween 80 facilitated the formation of smaller droplets in the emulsion, which increased the overall surface area for drug dissolution and absorption. The isopropyl alcohol solubilization process involved modifying the interfacial properties and improving the miscibility of the components in the formulation. Thus, the synergy among Capmul 337 MCM, Tween 80 and IPA may be the reason for the higher solubility. Building on the findings presented previously, Capmul MCM, Tween 80, and isopropyl alcohol were selected to develop the pseudo-ternary phase diagram necessary for formulating the Me system. This selection was made due to the exceptional solubilizing properties of these components, which are crucial for ensuring the effective delivery of IND within the Me-loaded CL formulation.

### 3.2. Pseudo-ternary phase diagram

**Fig. 2:**
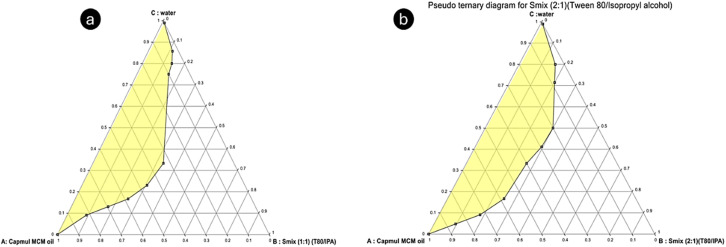
Pseudo ternary phase diagram for the (1 : 1) *S*_mix_ ratio (a) and (2 : 1) *S*_mix_ ratio (b).

**Fig. 3:**
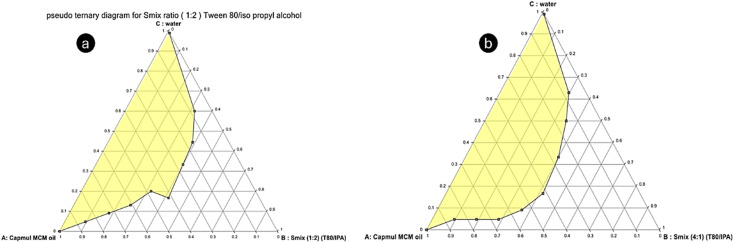
Pseudo-ternary phase diagram for the (1 : 2) *S*_mix_ ratio (a) and (4 : 1) *S*_mix_ ratio (b).

The pseudo-ternary phase diagram was created using the chosen components, including oil (Capmul MCM), *S*_mix_ (a blend of Tween 80 and isopropyl alcohol), and water. Various ratios of surfactant to co-surfactant (*S*_mix_) were employed, specifically 1 : 1, 2 : 1, 1 : 2, and 4 : 1, following the established procedure (Fig. 2 and 3). The resulting phase diagrams revealed the Me region, marked by a transparent phase, which was crucial for the subsequent preparation of Me. Upon analysis, it was observed that the *S*_mix_ ratios of 1 : 1, 1 : 2, and 4 : 1 exhibited a relatively narrow Me region, leading to their exclusion from further consideration. In contrast, the *S*_mix_ ratio of 2 : 1 demonstrated a larger Me region. Consequently, the pseudo-ternary phase diagram constructed with an *S*_mix_ ratio of 2 : 1 was employed as the basis for the subsequent development of the IND-loaded Me. Similar to the findings by Ning Wei *et al.*, this higher surfactant-to-*co*-surfactant ratio enhanced the stability and solubilization of IND, supporting its selection.^14^

### 3.3. Statistical data analysis using D-optimal mixture design

The objective for utilising the D optimal mixture design was to distinguish between batches originating from the microemulsion region and characterize the latter, rather than taking batches outside the microemulsion region. Conventional statistical designs, such as factorial and response surface designs, are insufficient for studying variables that are essentially components of a mixture. The D-optimal design allows the investigation of the interaction between different microemulsion components and their proportions in determining the desired dependent variables selected in this study. By applying the D-optimal mixture design, the observed responses for the formulated compositions were concurrently fitted to the response surface model. The data showed the observed responses (Y1 to Y3) for the IND Me-laden CLs. The response obtained from the D-optimal design is shown in Table 3.

**Table 3:** Batches of Me using the D-optimal design

Component 1 (X1)	Component 2 (X2)	Component 3 (X3)	Response1 (Y1)	Response 2 (Y2)	Response 3 (Y3)	
Run	A: oil	B: *S*_mix_	C: water	Globule size	Transmittance of Me diluted with water	Drug release after 6 h from Me-laden CLs prepared using the direct loading method
Units	mL	mL	mL	nm	(%)	(%)
1	0.229	0.535	0.235	110	11.1%	48.07%
2	0.322	0.572	0.104	120	12.2%	51.81%
3	0.323	0.350	0.326	140	14.3%	52.67%
4	0.437	0.350	0.212	166.5	16.6%	49.45%
5	0.550	0.350	0.100	170.9	17.8%	47.28%
6	**0.100**	**0.573**	**0.326**	**45**	**97.8%**	**44.5%**
7	0.322	0.572	0.104	120	75.2%	51.81%
8	0.436	0.463	0.100	180.1	18.2%	47.9%
9	**0.173**	**0.426**	**0.400**	**59.54**	**95.6%**	**49.02%**
10	**0.100**	**0.350**	**0.550**	**48.47**	**96.2%**	**46.9%**
11	0.229	0.535	0.235	110	11.1%	48.07%
12	**0.100**	**0.800**	**0.100**	**41.76**	**98.2%**	**44.7%**
13	**0.100**	**0.350**	**0.550**	**48.47**	**96.2%**	**46.9%**
14	**0.100**	**0.573**	**0.326**	**45**	**97.8%**	**44.5%**
15	0.323	0.350	0.326	120	14.7%	52.67%
16	**0.125**	**0.676**	**0.197**	**68.77**	**97.4%**	**44.7%**

#### 3.3.1. Data analysis of response (Y1): globule size

The globule size was determined for all preliminary developed batches using D-optimal design. The results showed a noteworthy variability (*p* < 0.0001) in the globule size, which clearly suggested that Y1 (dependent variable) was significantly affected by the selected (independent variable) X1, X2 and X3. The response (Y1) obtained at various levels of the three independent variables was analysed using multiple regression, resulting in a cubic polynomial equation.

The non-linear model produce for globule size was found to be significant with an F-value of 110.77 and *p* value of < 0.0001 with the *R*^2^ value of 0.9940. Therefore, among the formulated batches, only batches ME-6, 9, 10, 12, 13, 14, and 16 had a globule size of less than 70 nm. Batches ME-5 and 8 had the most significant negative effect, with globule sizes of 170.5 nm and 180 nm, respectively. This result was further confirmed from the regression analysis report that the Me batches with a lower oil phase and higher surfactant content showed better results. As the proportion of surfactant in the Me increased, the stabilization of the oil phase improved. A higher surfactant concentration allows more oil to be stabilized, leading to a decrease in globule size. This rise in the surfactant phase results in a notable reduction in globule size. The detailed ANOVA and regression analysis for globule size is shown in Table S4.†

#### 3.3.2. Data analysis of response (Y2): transmittance (%) of Me diluted with water

The transmittance (%) of the Me diluted with water was measured for the preliminary batches prepared using the D-optimal design. The response (Y2) was observed to vary from 11% to 98%. The response (Y2) clearly indicated that variation observed in Y2 (dependent variable) was significantly affected by the selected X1, X2 and X3. The response (Y2) observed at various levels of oil phase concentration (X1), surfactant mixture concentration (X2) and water (X3) (independent variable) was subjected to multiple regression analysis to generate a linear regression equation.

The microemulsion data revealed that batches ME-6, ME-9, ME-10, ME-12, ME-14, and ME-16 exhibit excellent stability and clarity, with high % transmittance values (∼95–98%) even after 10-fold dilution, indicating well-formed and stable microemulsions. Batches ME-4, ME-5, ME-8, and ME-15 show moderate stability, with transmittance values after dilution in the range of 14% to 18%. In contrast, ME-1, ME-2, ME-3, ME-7, and ME-11 exhibit significant reductions in transmittance upon dilution (as low as 11.1%), reflecting lower stability or incomplete microemulsion formation. Stability is strongly influenced by composition, with higher *S*_mix_ and balanced oil-to-water ratios promoting a superior performance, while oil-heavy or imbalanced formulations tend to reduce the stability and clarity. Overall, optimizing the *S*_mix_ and water content is critical for achieving stable microemulsions.

The linear model created for the response (Y2) showed an *F*-value of 6.63 and a *p*-value of 0.0057, demonstrating that the model was significant. *P*-values less than 0.05 suggested that the model terms were significant, whereas *p*-values greater than 0.1 indicated that the model terms were insignificant. The *R*^2^ value of 0.7682 implied a good signal to noise ratio. Thus, among the 16 batches, only batches ME-6, 9, 10, 12, 13, 14 and 16 exhibited significantly better transmittance (%), which was greater than 90%. In contrast, the other batches showed a negative effect on transmittance (%) exhibiting less than 80% transmittance.

The design space indicated that batches with more than 80% transmittance were o/w-type Mes, given that their external phase was miscible with water. In contrast, the batches that exhibited a negative response were not water-miscible, indicating they were w/o-type Mes. The batches located near the shaded region of the pseudo-ternary diagram were classified as w/o type due to their greater proportion of oil phase. Consequently, the levels of X1, X2, and X3 significantly influenced response 2 (Y2). The design space further suggested that higher levels of X2 and X3 would result in a positive transmittance when diluted with water, given that an increased level of X2 (*S*_mix_) effectively stabilizes X1 (oil phase). The detailed ANOVA table and regression analysis of transmittance are shown in Table S5.†

#### 3.3.3. Data analysis of response (Y3): drug release (%) in six hour from Me-laden CLs

The response (Y3)% drug release in 6 h from the Me-laden CLs prepared by the direct loading technique was observed for all the batches of Me and presented in Table S6.† The response (Y3) was observed in the range of 43% to 53%. The response (Y3) suggested the impact of X1, X2 and X3 (independent variable) at different levels with the variation observed in Y3 (dependent variable) in different batches. The fit summary suggested that the linear model is significant for the multiple regression analysis.

An equation with coded factors can be used to predict the response at specified levels of each factor. The linear model yielded an F-value of 17.40 with a *p*-value of 0.0001, demonstrating that the model was statistically significant. The *R*^2^ value was determined to be 0.8969. Therefore, batches ME-6, 9, 10, 12, 13, 14 and 16 implied significantly positive results. The drug release (%) after 6 h was less than 50%, which indicated a significantly positive and acceptable sustained drug release from the Me-loaded CLs prepared using the direct loading technique. The objective of the Me-loaded CLs was to achieve prolonged drug release. The response (Y3) suggested the impact of X2 (*S*_mix_) on the sustained drug release (%). As the concentration of X2 increases, a significant decline in (Y3) drug release (%) was observed. Thus, it was proposed that the surfactant traps the drug and oil globules within the micellar structure of the polymeric membrane of the *p*-HEMA hydrogel CLs. The detailed ANOVA table and regression analysis for drug release at 6 h are shown in Table S6.†

#### 3.3.4. Contour plots and surface response methodology

**Fig. 4:**
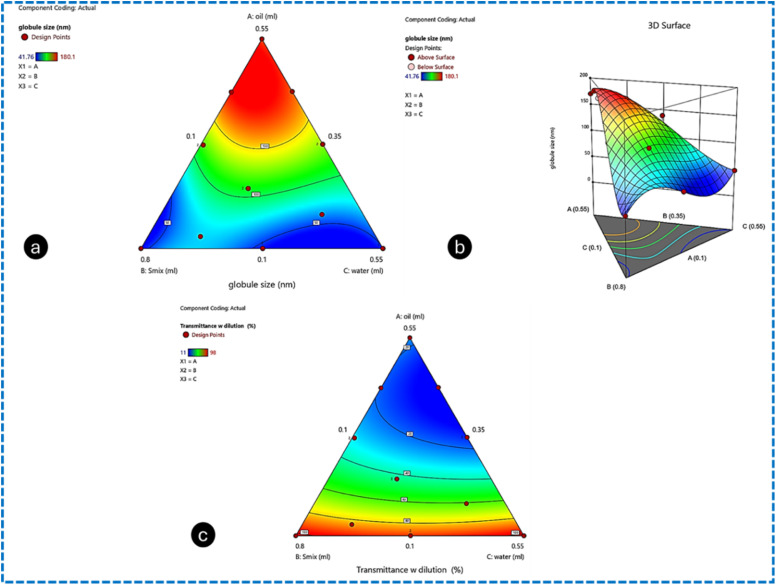
Contour plot for the response (Y1) (a), 3D plots of the response (Y1) (b), and contour plot for the response (Y2) (c).

**Fig. 5:**
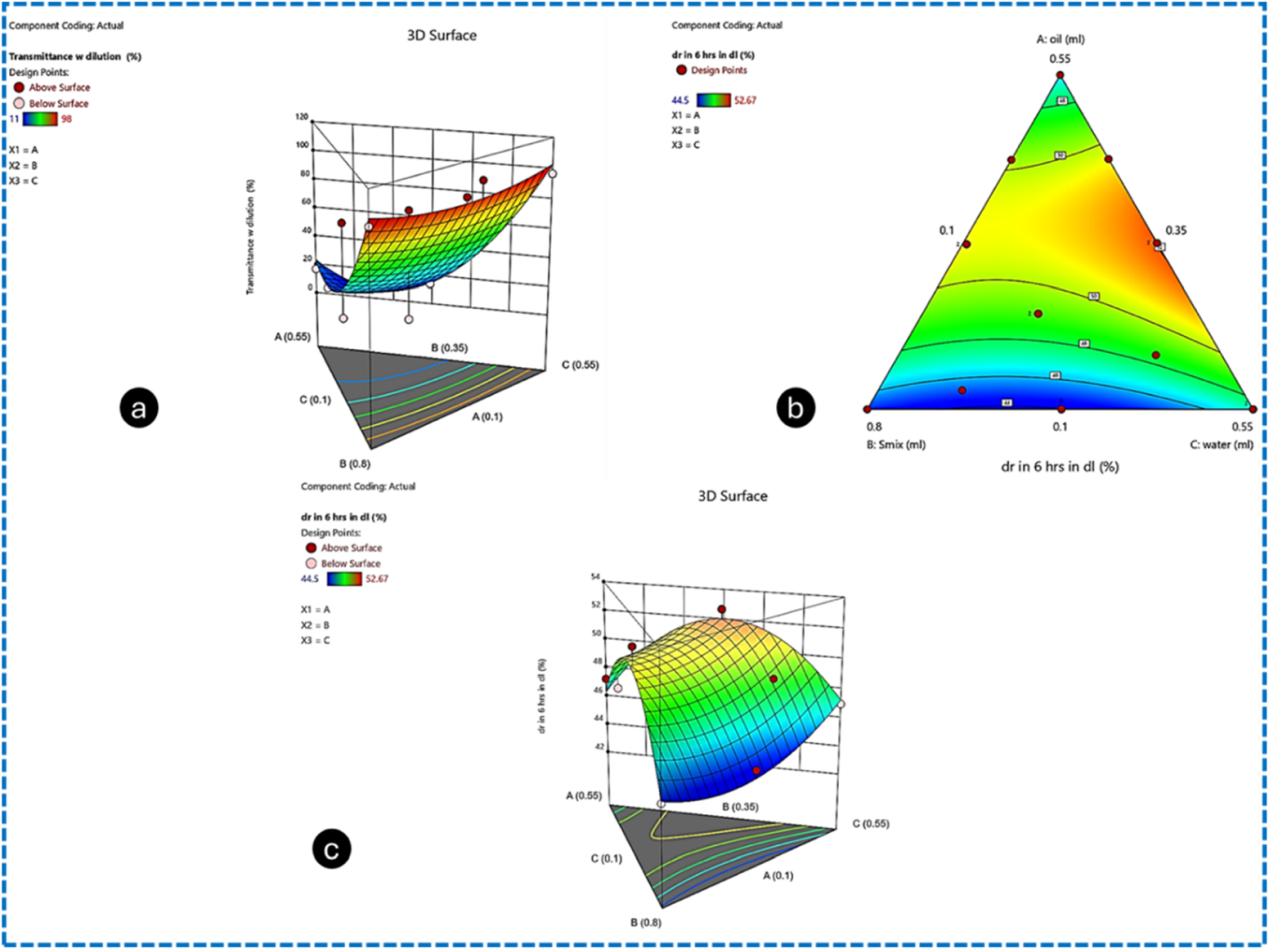
3D contour plot for the response (Y2) (a), 3D plots of the response (Y3) (b), and contour plot for the response (Y3) (c).

Contour plots were used to investigate the effect of independent variables on the globule size of the Me. The analysis showed that a target globule size of less than 70 nm can be achieved when the oil phase concentration (X1) is in the range of 0.102 mL to 0.112 mL, the surfactant phase (X2) is in the range of 0.371 mL and 0.709 mL, and the water content (X3) varies from 0.374 mL to 0.438 mL. The plots suggested that reducing the concentration of the oil phase (X1), while increasing the concentrations of the surfactant (X2) and water (X3) leads to a reduction in globule size from 170.9 nm to 45 nm. The desired globule size, ranging from 20 nm to 70 nm, is achievable with these specified concentrations of X1 and X2 (surfactant/cosurfactant mix). The contour plot and 3D surface plot for globule size are shown in Fig. 4(a and b), respectively. The contour plot was used to determine the impact of X1(oil), X2 (*S*_mix_) and X3 (water) on the response transmittance (%) (Y2). It was suggested that by using the contour plot design space that the targeted response value (Y2) (dependent variable) of transmittance (%) can be achieved by varying the concentration of the independent variables in the range of X1 (oil phase): 0.102 mL to 0.112 mL, X2 (*S*_mix_): 0.371 mL to 0.709 mL and X3 (water): 0.525 mL to 0.178 mL. Furthermore, the contour plot indicated that the transmittance (%) (Y2) will significantly improve by decreasing the oil concentration (X1) and increasing the amounts of surfactant (X2) and water (X3). However, the target transmittance (%) should remain above 80%. The contour and 3D surface plots for transmittance (%) are shown in Fig. 4c and 5a, respectively.

Using the Design Expert software version 12, the contour plot design space was generated, which revealed the impact of X1(oil), X2 (*S*_mix_) and X3 (water) on the dependent variable response (Y3) drug release (%) after 6 h from the Me-laden CLs prepared by the direct loading technique. The desired response Y3 can be achieved by keeping the concentration of X1 in range of, X2 and X3. Additionally, the desired drug release (%) within 6 h should not exceed 46%. This goal can be attained with the following concentrations: X1 (oil phase) in the range of 0.102 mL and 0.112 mL, X2 (*S*_mix_: surfactant/cosurfactant) in the range of 0.371 mL to 0.709 mL, and X3 (water) in the range of 0.525 mL to 0.178 mL. The contour plot and 3D surface plot for drug release at 6 h are shown in Fig. 5(b) and (c), respectively.

### 3.4. Optimization of Me using D-optimal design

**Fig. 6:**
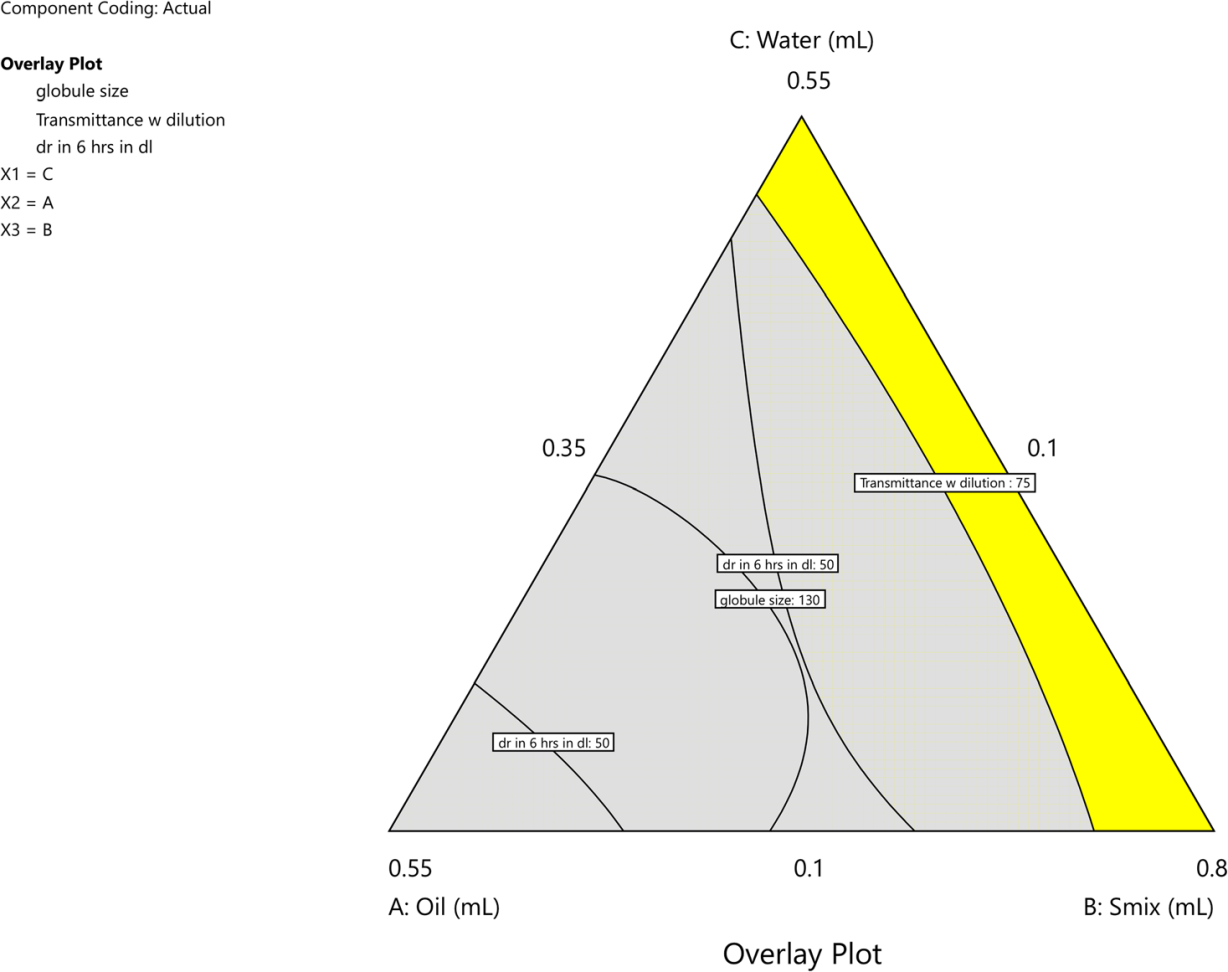
Overlay plot of the optimized batch.

Statistical analysis was conducted to examine the effect of X1 (oil), X2 (surfactant), and X3 (water) on Y1, Y2, and Y3. Considering all constraints, the D-optimal mixture design identified the optimal formulation with a desirability of 1.0. By selecting on the grid, the values of X1(oil): 0.104 mL, X2 (*S*_mix_): 0.795 mL and X3 (water): 0.1 mL for the desired globule size (nm) Y1: 39.57 nm, Y2 transmittance (%) of 100.59% and Y3 drug release (%) of 45.0603% can be achieved. The overlay plot for optimization of Me is shown in Fig. 6 and Table S7.†

### 3.5. Characterization of Me

#### 3.5.1. Dilution and transmittance

For the water-in-oil (w/o) Mes, dilution with deionized water typically results in phase separation and turbidity, indicating immiscibility. In contrast, when the oil-in-water (o/w) Mes is diluted with deionized water, they show no phase separation, confirming their miscibility and stability with the external phase. The transmittance (%) results indicated that the fabricated Me batches were transparent in their undiluted form. However, upon dilution with deionized water, batches ME-1, ME-2, ME-3, ME-4, ME-5, ME-7, ME-8, ME-11, and ME-15 exhibited a significant reduction in transmittance from 11.1% to 18.2%, suggesting the instability of these Mes. In contrast, batches ME-6, ME-9, ME-10, ME-12, ME-13, ME-14, and ME-16 maintained a transmittance (%) greater than 90% even after dilution, indicating their good stability. These stable batches were characterized by a lower oil content and an appropriate amount of surfactant and water, which contributed to their stability and high transmittance (%). Batches ME-6, ME-9, ME-10, ME-12, ME-13, ME-14, and ME-16 remained transparent upon dilution, indicating that these formulations were oil-in-water (o/w) Mes, given that their external phase was miscible with water. The remaining batches turned turbid or translucent, indicating their phase separation and instability upon dilution.

#### 3.5.2. Globule size, polydispersity index, ZP, and morphology

The key properties of Mes that predict their fate are globule size, PDI, and ZP. Thus, globule size, polydispersity index, and ZP were evaluated using the dynamic light scattering principle and a Zetasizer Nano ZS (Malvern Instruments, USA). The globule size, PDI, and ZP of the Mes are presented in Fig. S3(a–c),† respectively. Lower globule sizes indicate improved Me stability and enhanced clarity, which are particularly important for CL applications. The globule size ranged from 40.0 ± 5 nm to 180 ± 12 nm, which was within the acceptable range.^41^ The Me batches with a higher oil phase ratio and low surfactant ratio exhibited an increase in globule size, ranging from 120 ± 6.69 nm to 170.9 ± 10.82 nm. Conversely, the batches with a lower oil phase ratio and higher surfactant ratio exhibited a decrease in globule size. This was expected, given that a greater quantity of surfactant helped stabilize the oil phase, resulting in smaller globule sizes, often less than 70 nm.^42^ All the Me formulations showed globule sizes smaller than 200 nm, which did not affect the optical transmittance of the CLs after loading with the Me. The optimized microemulsion batch showed a globule size of 45.98 ± 8.49 nm (Fig. S4(a)†). Additionally, a PDI of less than 0.5 suggests a narrow and uniform distribution of globule sizes within the Me. Alternatively, a PDI greater than 0.5 suggests a poor size distribution, which can negatively affect the Me stability.^41^ The results showed that all the Me batches had a PDI of 0.5 or less, indicating their excellent stability. The optimized batch showed a PDI of 0.213, which is well below the 0.3 limit, and thus it was considered to have an acceptable size distribution and stable (Fig. S4(a)†). The ZP analysis provided insights into the stability of the Me, with higher ZP values reflecting improved electrostatic repulsion and stability. The results of the ZP analysis indicate that the Me globules may lack a charge, likely due to the use of non-ionic surfactants and cosurfactants.^43^ The ZP of all batches was nearly zero, confirming the absence of surface charge. The optimised batch showed a ZP of 0.657 ± 4.99 mV (Figure S4†(b)). This is attributed to the non-ionic nature of Tween 80 and isopropyl alcohol utilized in the formulations.^43^ Furthermore, the microstructure of the Mes tested using TEM revealed that the globules were spherical in shape, as depicted in Fig. S5.†

#### 3.5.3. Thermodynamic stability

The thermodynamic stability results indicated that all batches remained stable during the centrifugation test (Table S8†). Subsequently, these batches were subjected to a heating and cooling cycle. Batches ME-4 and ME-5 showed phase separation during this cycle, indicating destabilization of the Me, and therefore were not used in the freeze–thaw cycle. The other Me batches remained stable during the heating and cooling cycle, and thus subsequently tested in the freeze–thaw cycle. However, batches ME-3, ME-8, and ME-15 with a relatively equal amount of oil and *S*_mix_ exhibited cracking and phase separation, indicating their destabilization, and thus excluded from further evaluation. The ME-1, ME-2, ME-6, ME-7, ME-9, ME-10, ME-11, ME-12, ME-13, ME-14, and ME-16 batches did not display any signs of phase separation or cracking, and thus were considered thermodynamically stable under accelerated conditions.^29^ The findings also suggested that the Me batches with a higher oil phase ratio and equal or lower amounts of surfactant mixture exhibited destabilization and were deemed thermodynamically unstable.^44^ In contrast, the ME-1, ME-2, ME-6, ME-7, ME-9, ME-10, ME-11, ME-12, ME-13, ME-14, and ME-16 batches with a lower proportion of oil phase and a higher amount of surfactant showed excellent stability, with no phase separation or cracking.^44^ The thermodynamic stability assessment indicated that certain Me batches remained stable across centrifugation, heating-cooling, and freeze–thaw cycles, while others showed signs of phase separation and destabilization. Our findings are consistent with that by Yanchu Li *et al.*, who reported that Me batches prepared with an *S*_mix_ ratio of 1 : 4 (Pluronic F68 : PEG 200) demonstrated greater stability compared to those with a 1 : 6 ratio. Specifically, the ME-3 [1 : 6] and of ME-4 [1 : 6] batches showed phase separation under thermal stress, whereas the ME-1[1 : 4] batch passed all the tests, which is likely due to the stabilizing effect of Pluronic F68. This suggests that a higher proportion of Pluronic F68 enhances the stability by better encapsulating the oil globules and preventing phase separation.^12^ Similarly, the results reported by Ning Wei *et al.* demonstrated that Me batches with an increased surfactant concentration were more stable. In their study, batches with a 1 : 1 ratio of *S*_mix_ (Tween 20) failed under freeze–thaw cycles due to the insufficient surfactant needed to stabilize the oil droplets. However, batches ME-3 (1 : 2) and ME-4 (1 : 2), with a higher surfactant-to-oil ratio, remained thermodynamically stable.^14^ These findings reinforce the role of the surfactant concentration in maintaining stability, given that a higher *S*_mix_ ratio supports the formation of stable Mes under stress conditions. Thus, it can be concluded that the surfactant combination of Tween 80 and isopropyl alcohol, in a 2 : 1 ratio, was crucial in stabilizing the oil phase. Consequently, the Me formulations with a higher proportion of surfactant and a lower oil phase exhibited excellent stability.

### 3.6. Characterization of Me-loaded CLs

#### 3.6.1. Swelling study

The swelling study was performed on the control CLs (without drug) and IND-loaded CLs prepared by the direct loading technique and soaking technique. The swelling (%) of the CLs prepared by both the soaking and direct loading techniques is presented in Fig. S6(a and b),† respectively. The control CLs exhibited 98.67% ± 1.3% swelling without IND. The indomethacin-loaded CLs prepared by the direct loading technique showed the swelling of 57.14% ± 2.23%, while the same prepared by the soaking technique presented 78.57% ± 1.49% swelling. Thus, the indomethacin Me-laden CLs by the direct loading technique and soaking technique indicated positive results, which were greater than 85%. The swelling results of greater than or equal to 85% indicated that there is no significant alteration in the % swelling of CLs with the use of the IND Me system in the hydrogel CLs. The percentage swelling is directly related to their ion and oxygen permeability, as well as their dimensional changes.

#### 3.6.2. Transmittance study

Fig. S7(a and b) and Table S9† illustrates the transmittance (%) analysis of the CLs fabricated using the soaking and direct loading techniques. The transmittance (%) study was conducted on the control lenses without drug, which showed 98.80% ± 0.6% transmittance. In contrast, the IND-loaded lenses prepared *via* the direct loading and soaking techniques showed 81.3% ± 2.56% and 82.1% ± 3.01%. These results indicated that the drug loading in the hydrogel CLs may interfere with the optical property of the lenses. This might be due to the hydrophobic nature and poor solubility of the drug, leading to the precipitation of IND within the matrix of the pHEMA lenses.^35^ Moreover, the IND-loaded Me-laden contact lens formulated by the direct loading and soaking techniques showed a transmittance (%) greater than 95% due to the solubilization of IND in the Me core.

#### 3.6.3. Indomethacin leaching during sterilization study

The results indicate that after sterilization, the drug leached from the IND-loaded CLs was approximately 20.15% ± 0.5% for the lenses prepared by the soaking technique (IND-SL-CL) and 14.00% ± 2.38% for those prepared by the direct loading technique (IND-DL-CL), showing a higher degree of drug leaching compared to IND Me-laden CLs. Furthermore, the results showed that drug leaching from the Me-laden CLs prepared by the direct loading (1.21% ± 0.21%) technique was lower than that from the lenses prepared by the soaking technique (3.54% ± 0.58%). The soaking technique resulted in more drug leaching during the sterilization process compared to the direct loading technique. This might be due to the effective entrapment of the vesicles in the pHEMA matrix when the lenses were fabricated using the direct loading technique, whereas in the soaking technique, the vesicles are entrapped in aqueous channels.^45^ This suggests that the Me-laden CLs demonstrate better retention of the drug after sterilization.

#### 3.6.4. Drug content determination in CLs

The drug content assay (%) of IND-loaded CLs prepared by the direct loading technique was 79.39% ± 0.89%, while the IND Me-laden CLs showed an assay of greater than 97% ± 0.56%. The IND-loaded lenses prepared by the soaking technique displayed 20.05 ± 2.32 μg of drug, whereas the IND Me-laden lenses contained 27.0 ± 1.23 μg of drug. The Me-loaded CLs showed higher drug uptake than that of the free drug. The drug loading results for IND Me-laden CLs are consistent with the findings by Xu *et al.*, who reported the significantly enhanced uptake of bimatoprost when using an Me soaking solution compared to a conventional soaking solution. In their study, Xu *et al.* observed that the uptake of bimatoprost in CLs increased approximately two-fold when Mes were used, achieving loadings of 26.36 ± 2.57 μg, 46.36 ± 5.66 μg, and 61.39 ± 6.73 μg for Me concentrations of ME-25, ME-50, and ME-75, respectively. This enhancement was attributed to the higher partitioning of the Me system towards the CL matrix, enabling the more uniform dispersion and increased drug retention within the hydrogel structure. These results indicate that the drug content assay (%) of drug-loaded IND CLs was lower compared to the IND Me-laden lenses. The Me-laden lenses demonstrated higher drug loading efficiency than the conventional IND-loaded lenses. Additionally, the Me-laden lenses prepared by the soaking technique exhibited greater drug loading than that prepared by the direct loading technique.

#### 3.6.5. *In vitro* drug release of indomethacin Me-loaded CLs

Fig. S8(a)† illustrates the cumulative drug release (%) from the Me-loaded CLs. Fig. S8(b) and (c)† depict the cumulative drug release (%) from the CLs loaded with the drug using both the direct loading and soaking techniques. The conventional IND CLs (IND-DL-CL) prepared by the direct loading technique released the drug for 8 h, while the conventional IND CLs (IND-SL-CL) prepared by the soaking technique exhibited burst release, which released the drug for only 6 h. The results indicated that the drug release (%) from the Me-loaded CLs utilizing the direct loading technique was in the range of 44% ± 0.53% to 53% ± 0.59% during the initial 6 h, subsequently increasing to 74% ± 1.59% to 84% ± 1.89% after 24 h. Conversely, the drug release (%) from the CLs prepared through the soaking technique was in the range of 62.58% ± 1.56% to 97.64% ± 1.52% in the initial 6 h, and subsequently increased to 90% ± 1.76% to 98% ± 1.47% after 24 h. The results showed that the Me-laden CLs prepared by the direct loading technique released the drug for up to 28 h in a sustained and regulated nature. In contrast, the Me-laden CLs prepared by the soaking technique released almost 21.08% ± 1.00% to 31.69% ± 1.07% drug in first hour, and almost 90% drug in 24 h, which revealed burst release, followed by sustained release for an extended period.

#### 3.6.6. Ocular irritancy study in goat cornea

A study was performed to evaluate the ocular irritancy of the Me-loaded CLs to ensure the safety of CLs. The test formulation was assessed for irritation to the eye (cornea) using isolated goat corneas obtained from a local slaughterhouse. After exposure to the control or test solutions for 10 ± 1 min, the opacity of the isolated cornea was assessed by visual scoring and compared (Table 4).

**Table 4:** Result of corneal opacity score

Group	Solution	aCorneal opacity score
Negative control	0.9% w/v NaCl saline buffer	0.25 ± 0.50
Test formulation	Me-laden CLs prepared by soaking technique	0.75 ± 0.50
Positive control	100% ethanol	3.00 ± 0.82

a *n* = 3.

**Fig. 7:**
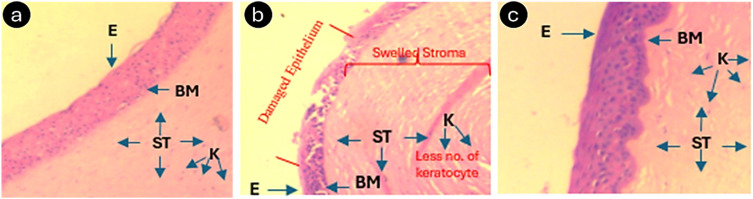
Representative H&E staining images of the cornea (negative control, NaCl, 0.9%) (a); positive control, ethanol (b); and test formulation, indomethacin (c). EP: Epithelium; BM: Bowman Layer; STR: Stroma.

The results indicated that 100% ethanol significantly (*p* < 0.05) induced opacity in the cornea on exposure for 10 min compared to that of the negative control (total of three corneas were used for each group). Further, the opacity induced by the solution used for preparing the Me-laden contact indicated an insignificant difference compared to the negative control (0.9% w/v NaCl). This clearly demonstrates that the solution did not induce any corneal irritation and appears to be safe with respect to opacity. Corneal opacity was seen in the positive control for irritancy. The negative control showed no opacity, which is nonirritant. The test formulation showed negligible and minimal opacity. This data revealed that the formulation is non-irritant given that it did not show any opacity. Following the irritancy study, the same eye was preserved in a 10% buffered formalin solution. The corneas were embedded in paraffin, sectioned using a microtome, and stained with hematoxylin. Examination of the cornea was conducted under a light microscope at 100× magnification. Fig. 7 presents the histopathological images of the cornea including A. negative control, B. positive control, and C. test formulation.

Fig. 7B depicts the effect of ethanol application, which caused epithelial cell disruption and disruption of the tight junctions between epithelial cells, leading to their detachment, shedding and swelling due to ethanol-induced osmotic stress. Damage to the epithelium compromises the protective barrier property of the cornea, and further loss of the epithelial layer leads to increased light scattering, contributing to corneal opacity. Keratocytes play a pivotal role in maintaining the extracellular matrix, similar to collagen and glycosaminoglycans, which provide corneal transparency and strength. Thus, the local exposure of ethanol to the cornea leads to the loss of keratocytes, eventually negatively affecting stromal repair. Further, damage to keratocytes can lead to stromal edema and disorganization of collagen fibers, reducing transparency and leading to opacity.^46,47^ In the negative control (Fig. 7a) and test groups (Fig. 7c), the appearance of a normal number of keratocytes, no signs of stroma swelling and intact epithelium indicated low possibility of opacity. The histology of the cornea exposed to the negative control showed a normal epithelium and stroma with keratocytes. Moreover, the histology of the cornea exposed to the positive control showed desquamation of the corneal epithelium, blood capillaries, and edematous stroma with inflammatory cells. In addition, the histology of the cornea exposed to the test formulation showed an intact corneal epithelium and stroma substantia propria with keratocytes. Therefore, this study suggests the compatibility of the formulation, which can be further tested in clinical trials.

## 4 Conclusions

This study effectively demonstrated the use of CLs loaded with an indomethacin Me for managing ocular inflammation. This promising approach for treating ocular inflammation enables sustained and controlled drug release, improved bioavailability, and reduced dosing frequency. The optimized Me system, utilizing Capmul MCM as the oil, Tween 80 as the surfactant, and isopropyl alcohol as a co-surfactant, demonstrated favourable Me properties, including a desirable globule size and transmittance (%) and enhanced stability. Furthermore, the Me-laden CLs exhibited indomethacin release for up to 28 h with the sustained drug delivery of IND. The ocular irritation studies revealed negligible irritation, indicating the safety and efficacy of the Me-laden CLs for prolonged drug delivery. The D-optimal mixture design confirmed the optimization of the Me system to improve the therapeutic effectiveness in managing ocular inflammation. Nevertheless, additional *in vivo* studies with animals wearing the CLs for prolonged durations (weeks) are required to evaluate conjunctival redness (hyperaemia), staining responses, and any changes in the cornea. Their potential clinical applications include treating conditions such as dry eye syndrome, uveitis, and post-surgical inflammation, providing an alternative to conventional eye drops. Additionally, their enhanced patient compliance and targeted delivery can make them a valuable option in ophthalmic therapeutics. From a commercial perspective, the microemulsion preparation process is robust, reproducible, and amenable to large-scale production, ensuring consistency and cost-effectiveness for industrial manufacturing. Similarly, the scalability of the contact lens manufacturing process has been progressed over the years with the advancement in polymer science, allowing the seamless integration of the optimized microemulsion formulation into standard manufacturing procedures. Given the extensive and growing global population of contact lens wearers, this formulation presents substantial market potential. The developed microemulsion-loaded contact lenses offer enhanced therapeutic delivery, while maintaining optimal lens comfort and transparency, providing a valuable competitive advantage in the ophthalmic market. These lenses align with the growing demand for advanced drug delivery systems in ophthalmic care, presenting opportunities for the pharmaceutical and medical device industries to develop novel, marketable ophthalmic treatments. The Me-loaded CLs show potential as an optimal approach for delivering BCS class II and IV drugs to treat ocular conditions.

## Data availability

Data can be made available from the corresponding authors upon request.

## Author contributions

Data curation and investigation: KP, YP, HKJ, MD; writing original draft and writing–review & editing: HKJ, YP; conceptualization, methodology, supervision, and writing–review & editing: KR and SS; and formal analysis: VRC, PM, AP, and DD.

## Conflicts of interest

The authors declare no conflicts of interest.

## Abbreviations

Indomethacin: IND
Hydroxyethyl methacrylate: HEMA
methacrylic acid: MAA
Ethylene glycol di-methacrylate: EGDMA
Capric acid: Captex 355
Glyceryl monocaprylate: Capmul MCM
Polyoxyethylene glycol: Lutrol F68
Caprylocaproyl polyoxyl-8 glycerides: Labrasol ALF
Oleoyl polyoxyl-6-glycerides: Labrafil M 1944 CS
Oil-in-water: o/w
Polydispersity index: PDI
Dynamic light scattering: DLS
Simulated tear fluid: STF
Lenses prepared by the soaking technique: IND-SL-CL
Lenses prepared by the direct loading technique: IND-DL-CL
Contact Lens: CL
Microemulsion: Me

## Supplementary Material

RA-015-D5RA01046B-s001
