# Supplementary material for: Indomethacin-incorporated microemulsion-laden contact lenses for improved ocular drug delivery and therapeutic efficacy
Source: RSC Adv. 2025 May 14;15(20):16110–24. doi: 10.1039/d5ra01046b (PMC12077302; doi:10.1039/d5ra01046b)
Supplement: RA-015-D5RA01046B-s001 [file RA-015-D5RA01046B-s001.pdf]

Supplementary Information (SI) for RSC Advances.
This journal is © The Royal Society of Chemistry 2025

Supplementary data

Indomethacin Incorporated Microemulsion-Laden Contact Lenses for Improved Ocular Drug Delivery and Therapeutic Efficacy

Kashvi Panchal[1], Yashkumar Patel[1], Harshilkumar Jani[1], Mittal Dalal[2], Vijay R Chidrawar[3], Deepanjan Datta[4], Popat Mohite[5], Abhijeet Puri[5], Ketan Ranch[1]* and Sudarshan Singh[6,7]*

[1]Department of Pharmaceutics and Pharmaceutical Technology, L. M. College of Pharmacy, Ahmedabad, Gujarat 380009, India [KP: kashvi38@gmail.com; KR: ranchketan@gmail.com; YP: yash.patel@lmcp.ac.in; HJ: harshil.jani@lmcp.ac.in]

[2]Department of Pharmacology, L. M. College of Pharmacy, Ahmedabad, Gujarat 380009, India [MD: mittal.dalal@lmcp.ac.in]

[3]School of Pharmacy and Technology Management, SVKM's Narsee Monjee Institute of Management Studies (NMIMS), Deemed-to-University, Green Industrial Park, TSIIC, Jadcherla, Hyderabad 509301, India [VRC: vijay.chidrawar@gmail.com]

[4]Department of Pharmaceutics, Manipal College of Pharmaceutical Sciences, Manipal Academy of Higher Education, Manipal, Karnataka 576104, India [DD: deepanjandtt@gmail.com]

[5]AETs St. John Institute of Pharmacy and Research, Palghar, Maharashtra 401404, India [PM: mohitepb@gmail.com; AP: abhijeetp@sjipr.edu.in]

[6]Office of Research Administration, Chiang Mai University, Chiang Mai 50200, Thailand

[7]Faculty of Pharmacy, Chiang Mai University, Chiang Mai 50200, Thailand [SS: sudarshan.s@cmu.ac.th]

***Correspondence**: sudarshan.s@cmu.ac.th; ranchketan@gmail.com

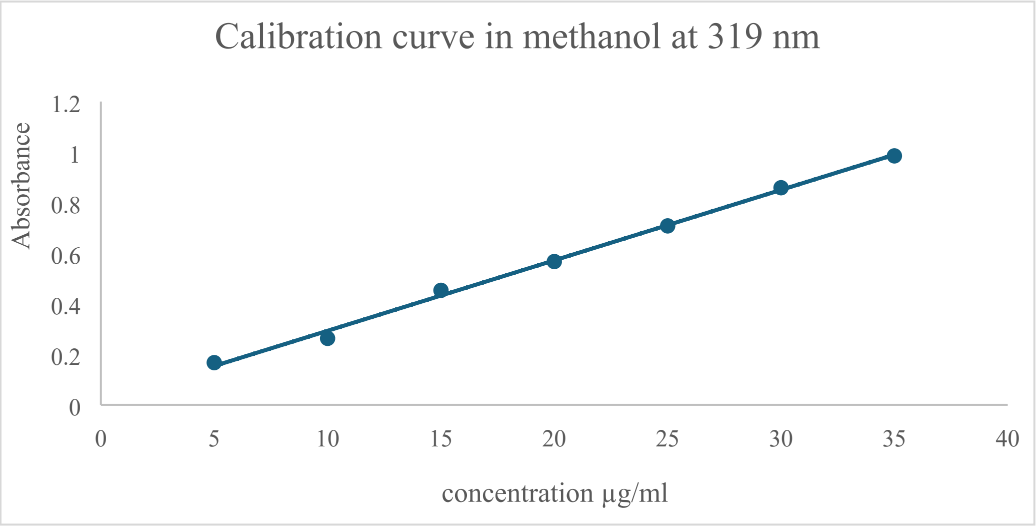

Figure S1. Calibration curve of IND in methanol using UV-Vis Spectrophotometer

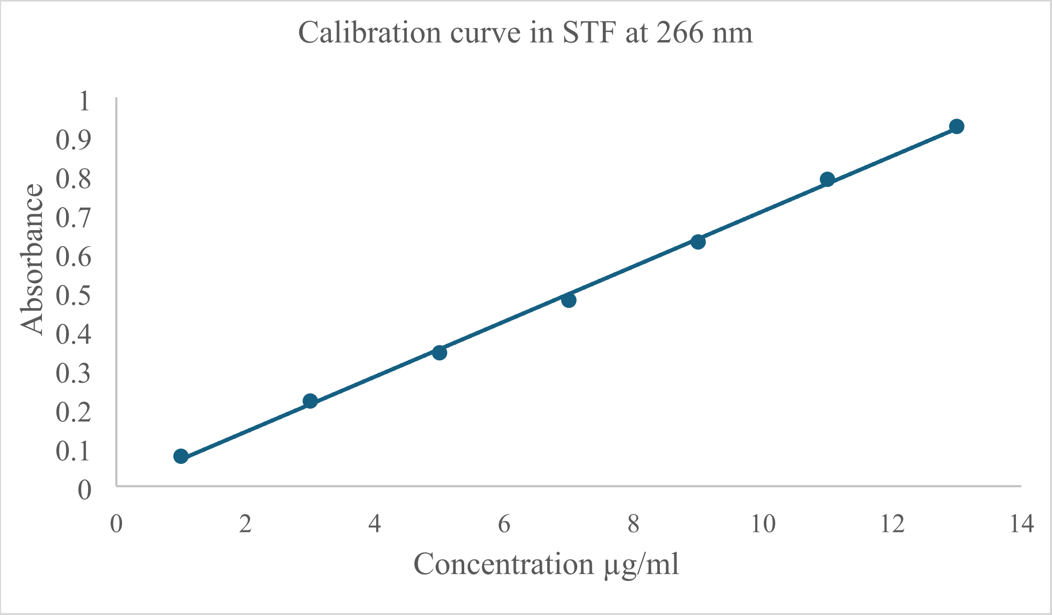

Figure S2. Calibration curve of IND in methanol using UV-Vis Spectrophotometer

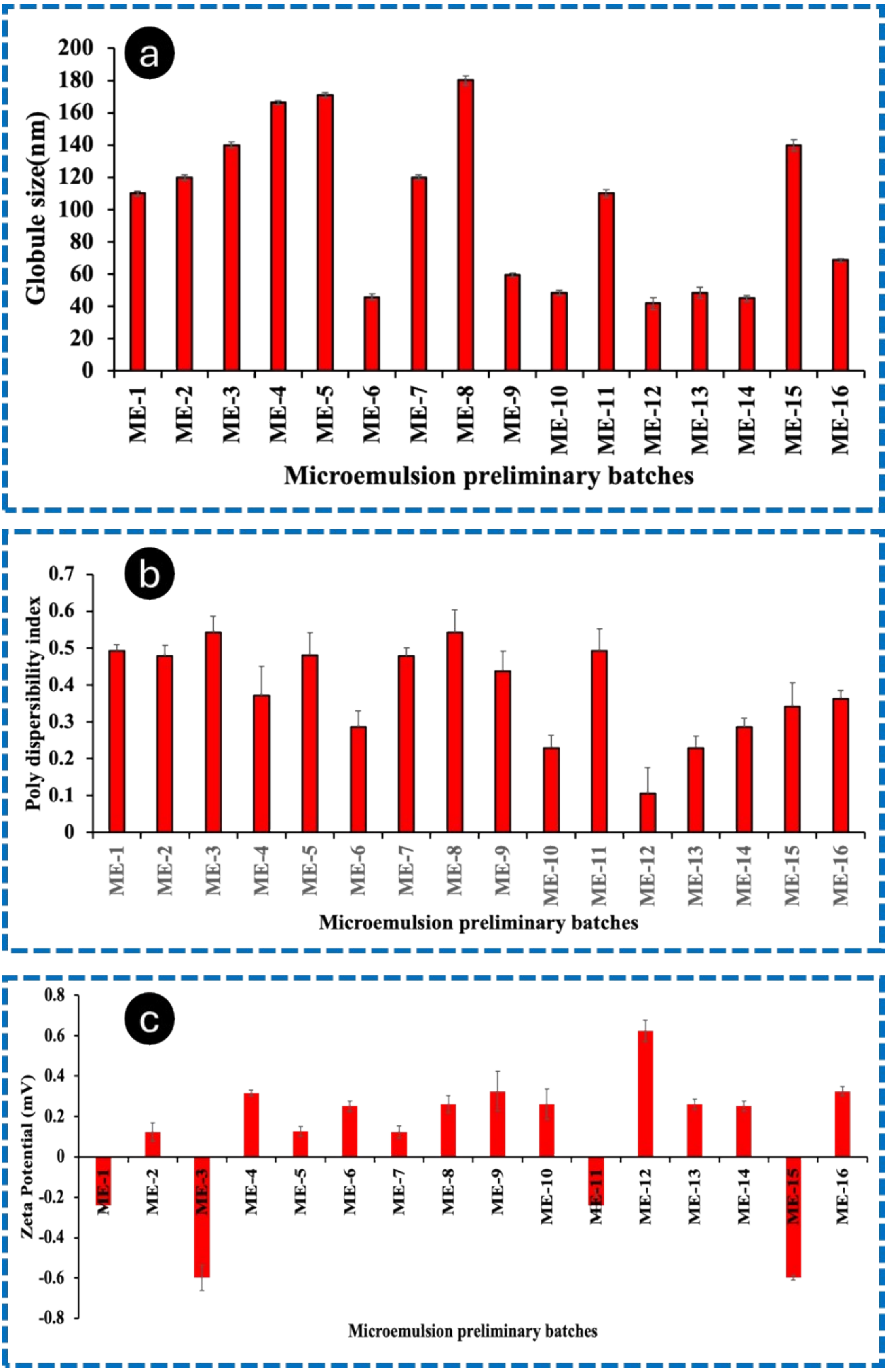

Figure S3. Globule size of Me (a), poly dispersibility index of Me (b), and ZP (c) of Me.

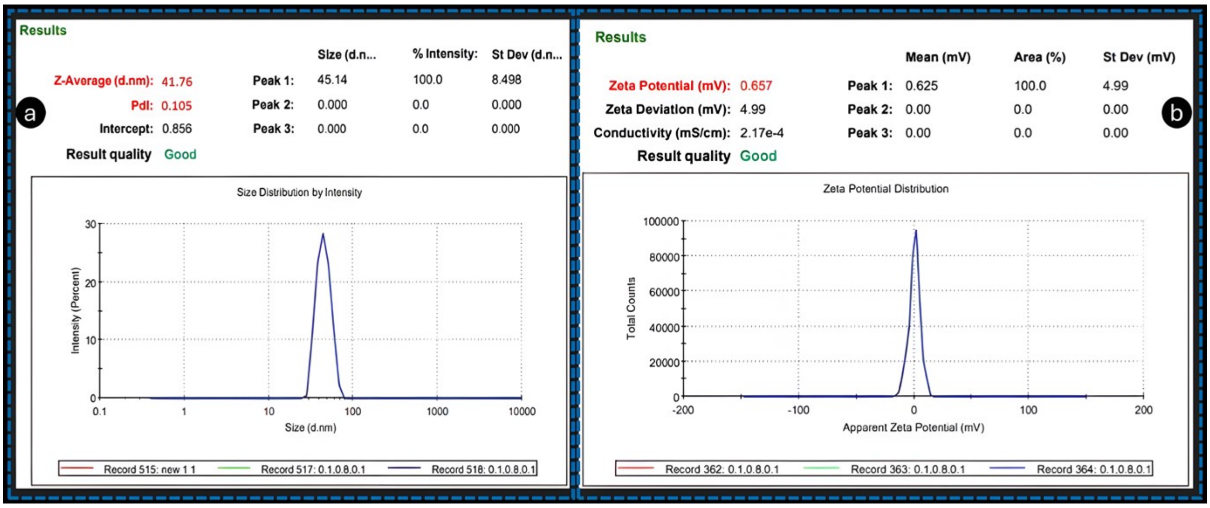

Figure S4. Globule size (a) and ZP (b) of optimized checkpoint batch.

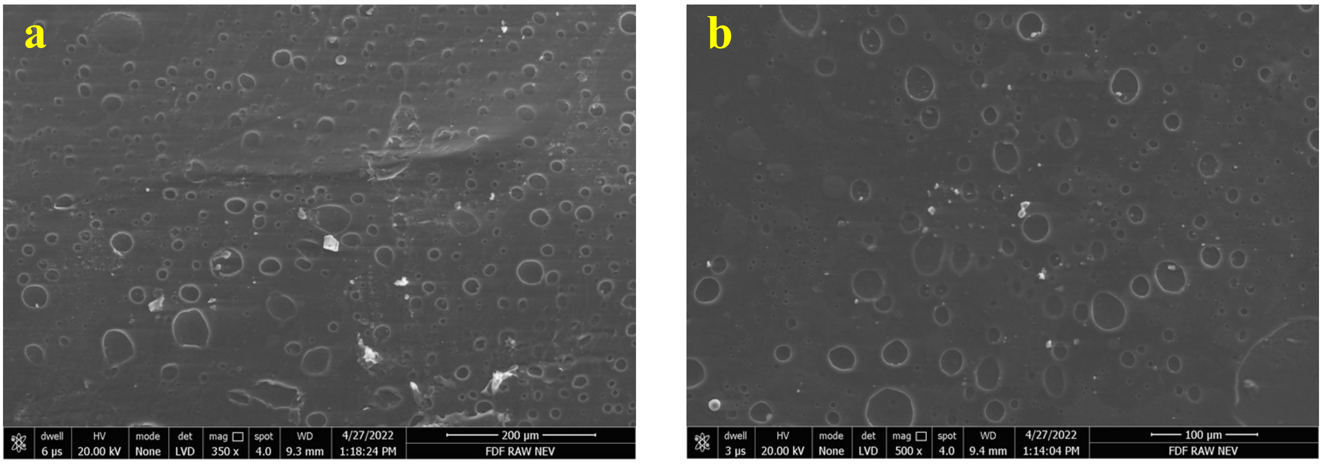

Figure S5. Transmission electron microscopy of optimised formulation at 350 x (b) and 500 x (b)

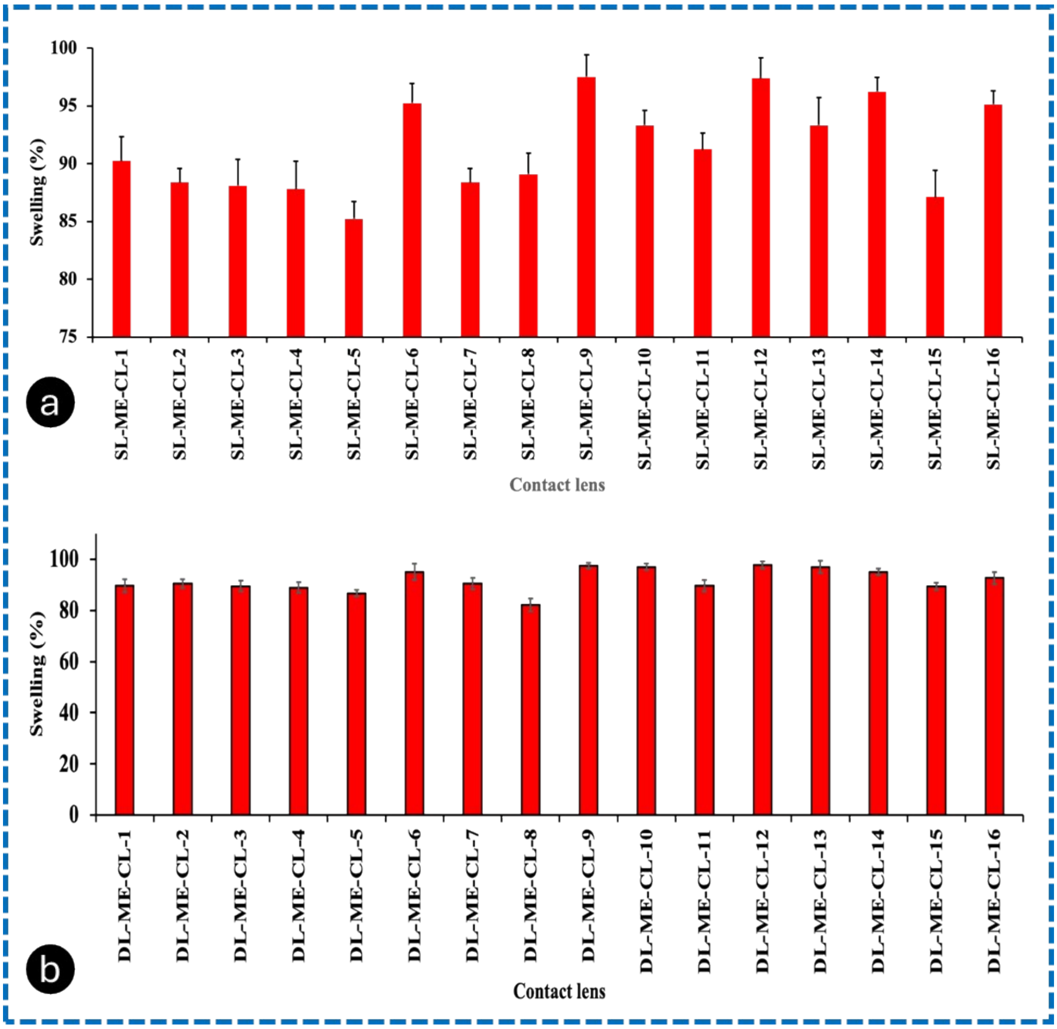

Figure S6. Swelling study (%) of CLs prepared by soaking technique (a), and direct loading technique (b).

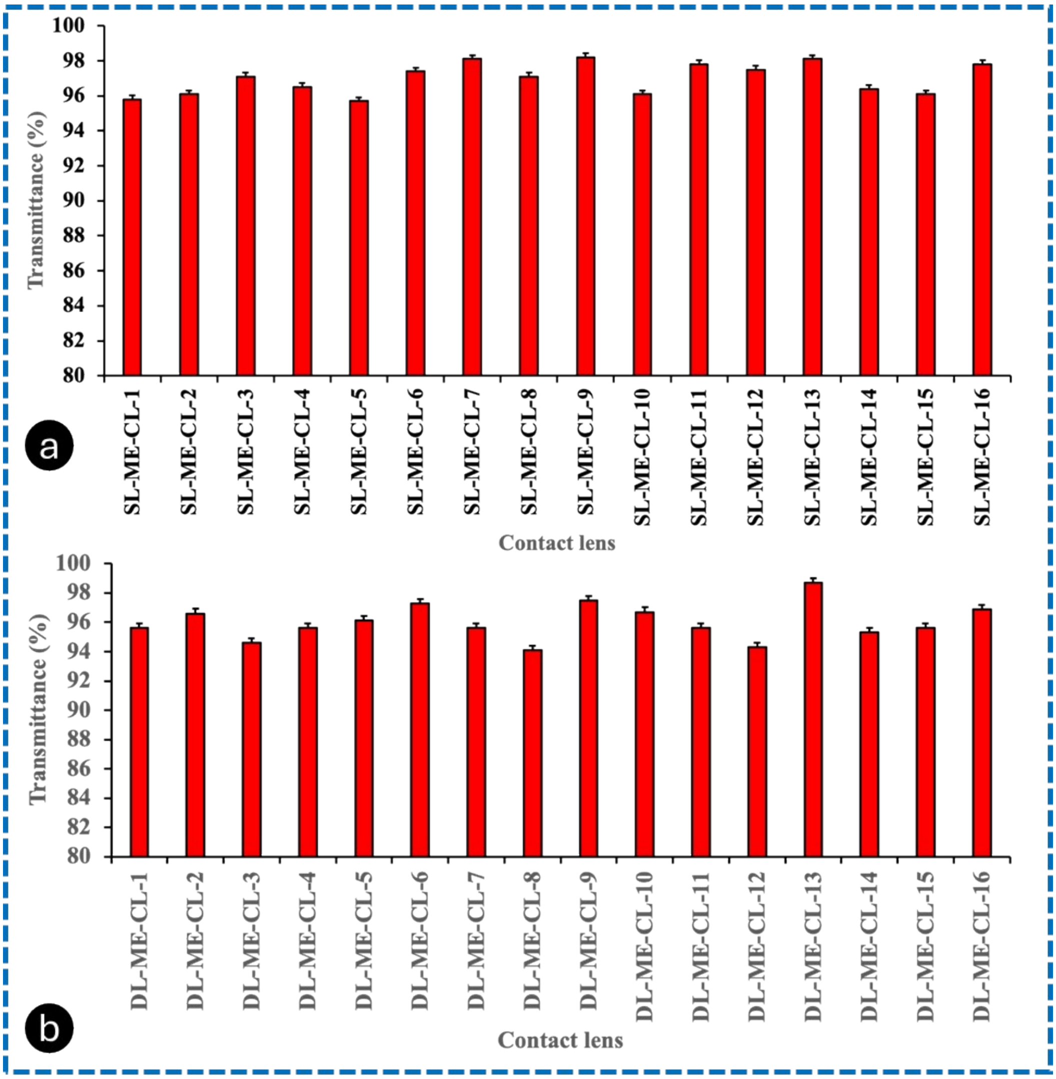

Figure S7. Transmittance (%) studies of CLs prepared by soaking technique (a), and direct loading technique (b)

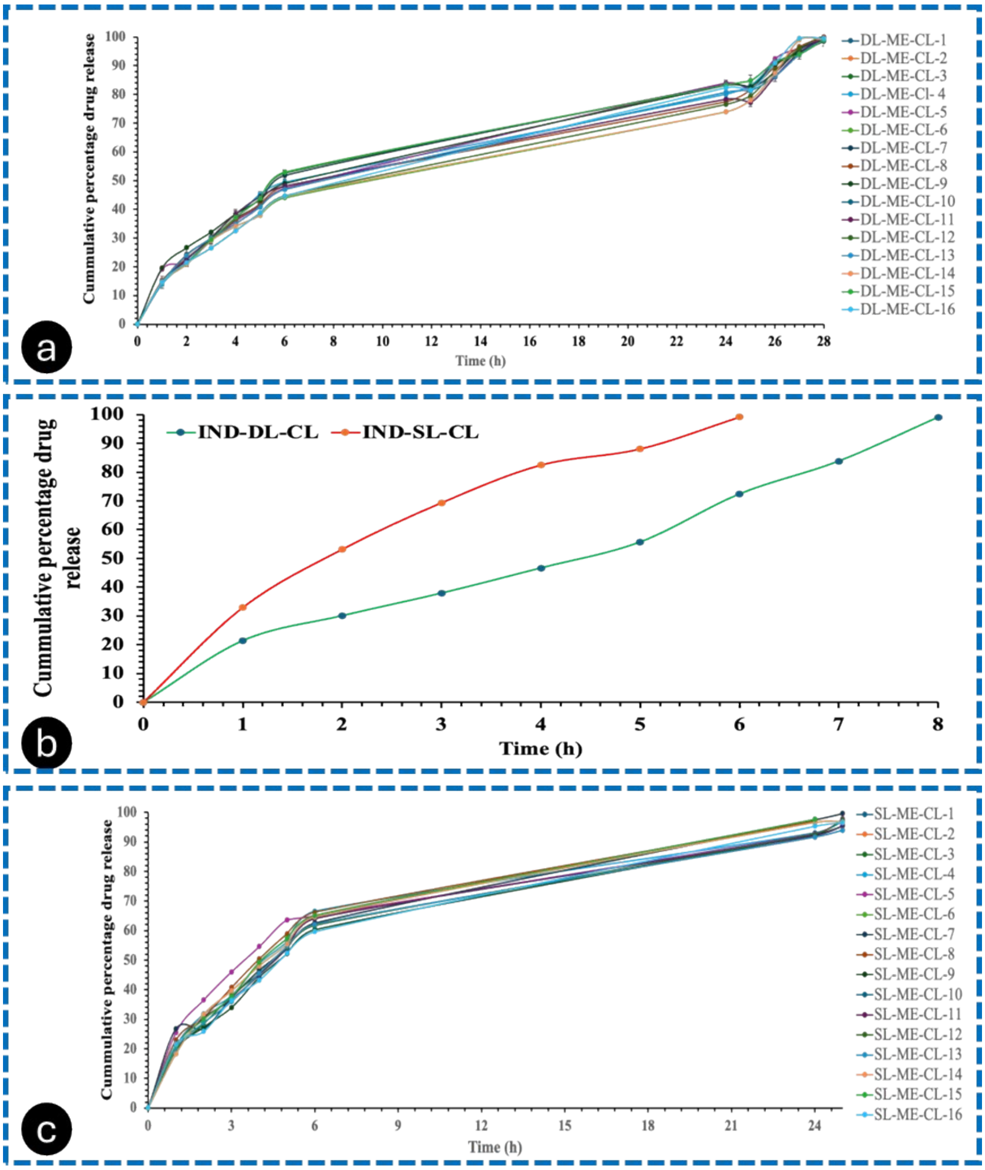

Figure S8. Cumulative drug release (%) from Me-laden CLs (direct loading technique) (batch DL-ME-1 to DL-ME-8) (a); cumulative drug release (%) from drug-loaded CLs fabricated by direct loading and soaking technique (b); and cumulative drug release (%) from Me-laden CLs (soaking method) (batch SL-ME-1 to SL-ME-8) (c).

Table S1. Calibration curve of IND in methanol using UV-Vis Spectrophotometer

Concentration (µg/ml)	Absorbance at 319nm			Average Absorbance	Standard Deviation (n=3)
5	0.163	0.177	0.172	0.167	0.004
10	0.260	0.260	0.270	0.263	0.005
15	0.429	0.520	0.411	0.453	0.058
20	0.573	0.568	0.561	0.567	0.006
25	0.749	0.697	0.678	0.708	0.036
30	0.861	0.928	0.790	0.859	0.006
35	0.935	0.985	0.983	0.985	0.001

Table S2. Calibration curve of IND in STF using UV-Vis Spectrophotometer

Concentration (µg/ml)	Absorbance at 266nm			Average Absorbance	Standard Deviation (n=3)
1	0.077	0.078	0.078	0.077	0.0005
3	0.219	0.218	0.220	0.219	0.001
5	0.343	0.342	0.344	0.343	0.001
7	0.478	0.477	0.479	0.478	0.001
9	0.628	0.627	0.629	0.628	0.001
11	0.788	0.789	0.79	0.789	0.001
13	0.925	0.924	0.926	0.925	0.001

Table S3. Batches of D-optimal mixture design

	**Oil (mL)**	**S_{mix} (2:1) (Tween 80/Iso propyl alcohol)**	**Water (mL)**
**ME-1**	0.229	0.535	0.235
**ME-2**	0.322	0.572	0.104
**ME-3**	0.323	0.350	0.326
**ME-4**	0.437	0.350	0.212
**ME-5**	0.550	0.350	0.100
**ME-6**	0.100	0.573	0.326
**ME-7**	0.322	0.572	0.104
**ME-8**	0.436	0.463	0.100
**ME-9**	0.173	0.426	0.400
**ME-10**	0.100	0.350	0.550
**ME-11**	0.229	0.535	0.235
**ME-12**	0.100	0.800	0.100
**ME-13**	0.100	0.350	0.55
**ME-14**	0.100	0.573	0.326
**ME-15**	0.323	0.350	0.326
**ME-16**	0.125	0.676	0.197

Table S4. ANOVA table and regression analysis for response (Y1)

Source	Sum of squares	df	Mean square	F-value	p-value	
**Model**	36011.36	9	4001.26	110.77	< 0.0001	significant
**Linear Mixture**	33746.59	2	16873.29	467.11	< 0.0001	
**AB**	148.09	1	148.09	4.10	0.0893	
**AC**	481.23	1	481.23	13.32	0.0107	
**BC**	0.0091	1	0.0091	0.0003	0.9879	
**ABC**	308.55	1	308.55	8.54	0.0265	
**AB(A-B)**	739.97	1	739.97	20.49	0.0040	
**AC(A-C)**	149.14	1	149.14	4.13	0.0884	
**BC(B-C)**	576.12	1	576.12	15.95	0.0072	
**Residual**	216.73	6	36.12			
**Lack of fit**	16.73	1	16.73	0.4184	0.5463	not significant
**Pure error**	200.00	5	40.00			
**Cor total**	36228.09	15				
**Regression analysis of response (Y1)**						
**R^2**	**0.9940**					
**Equation**						
**Full model**	Y1 = 170.39 X1 + 41.28 X2 + 48.30 X3 + 49.91 X1X2 + 81.19 X1X3 - 0.353X2X3 + 484.90 X1X2X3 + 356.29 X1X2(X1-X2) + 137.92 X1X3(X1-X3) + 291.28 X2X3(X2-X3)					
**Reduced model**	Y1 = 170.39 X1 + 41.28 X2 + 48.30 X3+ 81.19 X1X3+ 484.90 X1X2X3 + 356.29 X1X2(X1-X2) + 291.28 X2X3(X2-X3)					

Table S5. ANOVA table and regression analysis of response (Y2)

**Source**	**Sum of squares**	**df**	**Mean square**	**F-value**	**p-value**	
**Model**	20046.53	5	4009.31	6.63	0.0057	significant
**Linear mixture**	16147.29	2	8073.64	13.35	0.0015	
**AB**	1366.02	1	1366.02	2.26	0.1638	
**AC**	3348.82	1	3348.82	5.54	0.0404	
**BC**	106.96	1	106.96	0.1769	0.6830	
**Residual**	6047.26	10	604.73			
**Lack of fit**	4062.76	5	812.55	2.05	0.2252	not significant
**Pure error**	1984.50	5	396.90			
**Cor Total**	26093.79	15				
**Regression analysis of response (Y2)**						
**R^2**	**0.7682**					
**Equation**						
**Full model**	(Y2) = 23.66 X1 + 102.73 X2 + 102.55 X3 – 130.69 X1X2 -194.64 X1X3 – 35.42 X2X3					
**Reduced model**	(Y2) = 23.66 X1 + 102.73 X2 + 102.55 X3-194.64 X1X3					

Table S6. ANOVA Table and regression analysis for response (Y3)

Source	Sum of squares	df	Mean square	F-value	p-value	
**Model**	111.65	5	22.33	17.40	0.0001	significant
**Linear mixture**	45.77	2	22.89	17.83	0.0005	
**AB**	31.56	1	31.56	24.59	0.0006	
**AC**	38.74	1	38.74	30.18	0.0003	
**BC**	5.80	1	5.80	4.52	0.0594	
**Residual**	12.84	10	1.28			
**Lack of Fit**	12.84	5	2.57			Non-significant
**Pure error**	0.0000	5	0.0000			
**Cor total**	124.49	15				
**Regression analysis of response (Y3)**						
**R^2**	**0.8969**					
**Equation**						
**Full model**	(Y3) = 46.40 X1 + 44.84 X2 + 47.17 X3 + 19.87 X1X2 + 20.93 X1X3 – 8.25 X2X3					
**Reduced model**	(Y3) = 46.40 X1 + 44.84 X2 + 47.17 X3+ 19.87 X1X2 + 20.93 X1X3					

Table S7: Coefficient table

Response	A: oil	B: S_{mix}	C: water	AB	AC	BC	ABC
**globule size (Y1)**	**170.39**	**41.28**	**48.30**	49.91	**81.19**	-0.353	**484.9**
**p-values**	**< 0.0001**	**< 0.0001**	**< 0.0001**	0.08	**0.010**	0.987	**0.026**
**Transmittance (%)(Y2)**	**23.65**	**102.72**	**102.54**	-130.68	**-194.63**	-35.42	-
**p-values**	**0.0015**	**0.0015**	**0.0015**	0.16	**0.040**	0.68	-
**Drug release (%) (Y3)**	**46.40**	**44.84**	**47.17**	**19.86**	**20.93**	-8.24	-
**p-values**	**0.0005**	**0.0005**	**0.0005**	**0.0006**	**0.0003**	0.059	-

Table S8. Result of thermodynamic stability study

**Batch no.**	**Centrifugation test**	**Heating cooling cycle**	**Freeze Thaw cycle**
ME-1	Stable	Stable	Stable
ME-2	Stable	Stable	Stable
ME-3	Stable	Stable	**Cracking**
ME-4	Stable	**Phase separation**	-
ME-5	Stable	**Phase separation**	-
ME-6	Stable	Stable	Stable
ME-7	Stable	Stable	Stable
ME-8	Stable	Stable	**Phase separation**
ME-9	Stable	Stable	Stable
ME-10	Stable	Stable	Stable
ME-11	Stable	Stable	Stable
ME-12	Stable	Stable	Stable
ME-13	Stable	Stable	Stable
ME-14	Stable	Stable	Stable
ME-15	Stable	Stable	**Cracking**
ME-16	Stable	Stable	Stable

Table S9. Transmittance study (%) and dilution test of batches of microemulsion

**Batch no.**	**Components**			**Transmittance (%) of microemulsion**	**Transmittance (%) after 10 times dilution**
	Oil	S_{mix}	Water	(%)	(%)
**ME-1**	0.229	0.535	0.235	91.2	11.1
**ME-2**	0.322	0.572	0.104	90.2	12.2
**ME-3**	0.323	0.350	0.326	89.8	14.3
**ME-4**	0.437	0.350	0.212	92.6	16.6
**ME-5**	0.550	0.350	0.100	91.4	17.8
**ME-6**	**0.100**	**0.573**	**0.326**	**97.3**	**97.8**
**ME-7**	0.322	0.572	0.104	90.3	75.2
**ME-8**	0.436	0.463	0.100	93.2	18.2
**ME-9**	**0.173**	**0.426**	**0.400**	**97.5**	**95.6**
**ME-10**	**0.100**	**0.350**	**0.550**	**96.7**	**96.2**
**ME-11**	0.229	0.535	0.235	91.2	11.1
**ME-12**	**0.100**	**0.800**	**0.100**	**98.0**	**98.2**
**ME-13**	**0.100**	**0.350**	**0.550**	**96.7**	**96.2**
**ME-14**	**0.100**	**0.573**	**0.326**	**97.2**	**97.8**
**ME-15**	0.323	0.350	0.326	89.8	14.7
**ME-16**	**0.125**	**0.676**	**0.197**	**97.1**	**97.4**
